# Expression of Dystroglycanopathy-Related Enzymes, POMGNT2 and POMGNT1, in the Mammalian Retina and 661W Cone-like Cell Line

**DOI:** 10.3390/biomedicines13112759

**Published:** 2025-11-11

**Authors:** Cristina Quereda, Violeta Gómez-Vicente, Mercedes Palmero, José Martín-Nieto

**Affiliations:** 1 Departamento de Fisiología, Genética y Microbiología, Facultad de Ciencias, Universidad de Alicante, Campus Universitario San Vicente, P.O. Box 99, E-03080 Alicante, Spain; 2 Departamento de Óptica, Farmacología y Anatomía, Facultad de Ciencias, Universidad de Alicante, E-03080 Alicante, Spain; 3 Instituto de Investigación Sanitaria y Biomédica de Alicante (ISABIAL), E-03080 Alicante, Spain; 4 Instituto Multidisciplinar para el Estudio del Medio ‘Ramón Margalef’, Universidad de Alicante, E-03080 Alicante, Spain

**Keywords:** POMGNT2, POMGNT1, dystroglycan, dystroglycanopathies, retinal cells

## Abstract

**Background.** Dystroglycanopathies (DGPs) constitute a set of recessive, neuromuscular congenital dystrophies that result from impaired glycosylation of dystroglycan (DG). These disorders typically course with CNS alterations, which, alongside gradual muscular dystrophy, may include brain malformations, intellectual disability and a panoply of ocular defects. In this process, the protein products of 22 genes, collectively dubbed DGP-associated genes, directly or indirectly participate sequentially along a complex, branched biosynthetic pathway. POMGNT2 and POMGNT1 are two enzymes whose catalytic activity consists of transferring the same substrate, a molecule of *N*-acetylglucosamine (GlcNAc) to a common substrate, the *O*-mannosylated α subunit of DG. Despite their presumptive role in retinal homeostasis, there are currently no reports describing their expression pattern or function in this tissue. **Purpose.** This work focuses on POMGNT2 and POMGNT1 expression in the mammalian retina, and on the characterization of their distribution across retinal layers, and in the 661W photoreceptor cell line. **Methods.** The expression of POMGNT2 protein in different mammalian species’ retinas, including those of mice, rats, cows and monkeys, was assessed by immunoblotting. Additionally, POMGNT2 and POMGNT1 distribution profiles were analyzed using immunofluorescence confocal microscopy in retinal sections of monkeys and mice, and in 661W cultured cells. **Results.** Expression of POMGNT2 was detected in the neural retina of all species studied, being present in both cytoplasmic and nuclear fractions of the monkey and mouse, and in 661W cells. In the cytoplasm, POMGNT2 was concentrated in the endoplasmic reticulum (ER) and/or Golgi complex, depending on the species and cell type, whereas POMGNT1 accumulated only in the Golgi complex in both monkey and mouse retinas. Additionally, both proteins were present in the nucleus of the 661W cells, concentrating in the euchromatin and heterochromatin, as well as in nuclear PML and Cajal bodies, and nuclear speckles. **Conclusions.** Our results are indicative that POMGNT2 and POMGNT1 participate in the synthesis of *O*-mannosyl glycans added to α-dystroglycan in the ER and/or Golgi complex in the cytoplasm of mammalian retinal cells. Also, they could play a role in the modulation of gene expression at the mRNA level, which remains to be established, in a number of nuclear compartments in transformed retinal neurons.

## 1. Introduction

Central nervous system (CNS) development and function requires a proper *O*-mannosyl glycosylation of dystroglycan (DG) [1,2,3,4,5,6]. In humans, deficiencies in this complex process are causative of a set of recessive genetic disorders referred to as dystroglycanopathies (DGPs), or muscular dystrophies-dystroglycanopathies (MDDGs) according to the OMIM database classification. These are congenital neuromuscular dystrophies with associated CNS abnormalities featuring progressive muscle atrophy and often accompanied by brain malformation, cognitive impairment and a wide variety of ocular anomalies, and which offer a short life expectancy in the most serious cases [7,8,9,10,11]. The clinical manifestations of DGPs may display a broad range of severity according to which they are designated, from the most to the least severe forms, as Walker-Warburg syndrome, muscle-eye-brain disease, Fukuyama congenital muscular dystrophy, congenital muscular dystrophies of intermediate severity, and limb-girdle muscular dystrophies [8,9,10].

DG is encoded by the *DAG1* gene and was originally identified in the brain as a predominant plasma-membrane glycoprotein with the capacity to bind laminin [12,13,14], the main constituent component of the basal lamina within the extracellular matrix (ECM) [15]. The primary structure of DG is highly conserved across vertebrate species and is expressed in a broad range of fetal and adult tissues including, but not limited to muscle, neural, adipose, epithelial, endothelial and blood tissues, all of which share the property of cells being in direct contact with basement membranes, and with DG being most abundant especially in the skeletal muscle and brain [13,14,16,17,18,19]. In its maturation process the DG polypeptide undergoes post-translational autocatalytic cleavage to yield its two constituent subunits, α-DG and β-DG, which remain non-covalently associated thereafter [13,20,21,22]. The α-DG subunit is an extracellular membrane-associated polypeptide composed of two N- and C-terminal globular domains separated by a mucin-like region heavily glycosylated with *O*-linked sugar chains, predominantly *O*-mannosyl glycans [13,23,24,25]. By contrast, the β DG subunit is an integral transmembrane polypeptide lacking *O*-glycosylation sites [13,22]. DG constitutes a core component of the dystrophin-glycoprotein complex (DGC), which establishes a structural and functional link between ECM proteins, such as laminin and proteoglycans, and the actin cytoskeleton through dystrophin in both skeletal muscle and non-muscular tissues [5,23,26]. In this complex, α-DG is responsible for the cells’ attachment to the ECM via its interaction with ECM components, including laminin, agrin, perlecan and biglycan in muscle and neural tissues, neurexin and slit in the brain, and pikachurin in the retina, a specificity conferred by the complex *O*-mannosyl glycans attached to the α-DG mucin-like domain [27,28,29,30]. Additionally, within the DGC the cytoplasmic domain of β-DG is anchored to the actin cytoskeleton through its interaction with dystrophin or utrophin, the latter being a dystrophin paralog expressed in non-muscle tissues and at the neuromuscular junction [31,32].

Loss of function mutations in DGP-associated genes result in impairments in the glycosylation of α-DG, which diminishes or abolishes α-DG binding affinity to its extracellular ligands. To date, a total of 22 genes have been identified that encode, in addition to DG itself (*DAG1* gene), a set of known or putative glycosyltransferases and other enzymes, most of which reside in the endoplasmic reticulum (ER) or the Golgi complex. These proteins are directly or indirectly involved in the addition of *O*-mannosyl glycans to the mucin-like region of α-DG by sequentially acting along a branched, complex glycosylation pathway [30,32,33,34,35]. Especially in the most severe forms of DGPs (see above), mutational defects in such pathway lead to the development of ocular anomalies including retinal malformations and dysplasias, as well as ophthalmic pathologies such as cataracts, severe myopia and/or glaucoma, all of which cause a partial or total loss of vision [4,36]. In the retina, the interaction between DG and pikachurin is essential for the proper formation and function of ribbon synapses connecting photoreceptors (cones and rods) to their postsynaptic, bipolar and horizontal cells within the outer plexiform layer (OPL) [29]. In addition, DG produced by Müller glia interacts with ECM proteins to facilitate the assembly of the inner limiting (basement) membrane, which separates the neural retina from the vitreous humor [37,38].

Protein *O*-linked-mannose β-1,4-*N*-acetylglucosaminyltransferase 2 (POMGNT2) and β-1,2-*N*-acetylglucosaminyltransferase 1 (POMGNT1) are two enzymes whose catalytic activity consists of transferring a molecule of *N*-acetylglucosamine (GlcNAc) to *O*-mannose units linked to the α-DG mucin-like domain, and hence sharing the same substrate [39,40]. It has been shown that POMGNT2 exhibits catalytic activity on a set of particular *O*-mannosylated residues of α-DG, acting in a selective fashion, while POMGNT1 is capable of potentially glycosylate any *O*-mannosylated residue of α-DG [41]. To date, these subpathways have been characterized exclusively for the specific *O*-mannose residues that are initially added by the POMT1/POMT2 heterodimer to Ser/Thr residues within the α-DG mucin-like domain. In this context, POMGNT2, presumably located in the ER, catalyzes the initial step of the biosynthetic subpathway that generates the α-DG core *O*-mannosyl glycan structure called M3 [39,40,42,43,44,45]. This structure is essential for the cell’s attachment to the ECM by providing a link between α-DG and its binding-protein partners in the ECM [46,47]. A defective core M3 glycosylation appears to be a common factor between DGPs and an increased metastasis in some carcinomas, as well as between DGPs and various forms of aberrant neuronal migration and axon guidance in mammals [47,48,49,50,51,52,53,54,55,56,57,58,59]. On the other hand, POMGNT1, presumably residing in the Golgi complex, initiates the biosynthetic subpathway leading to building of the α-DG core *O*-mannosyl glycan structures M1 and M2 [39,40,57,60,61,62,63].

To elucidate the in vivo function(s) of POMGNT2 and POMGNT1, several mouse models have been generated and extensively characterized by means of *Pomgnt2* and *Pomgnt1* inactivation to yield knockout (KO) mice deficient in POMGNT2 [44,64] or POMGNT1 [38,46,65,66,67,68,69,70,71,72,73,74,75,76,77] proteins. In all these models, mice exhibited multiple defects, both structural and functional, in the muscle, brain and eye, together with associated α-DG glycosylation defects recapitulating those detected in patients with DGPs. In addition, it was noticeable that *Pomgnt2* KO mice displayed an aberrant neuronal migration in the developing brain [44,64]. Nevertheless, the precise role of POMGNT2 in the adult visual system remains largely unknown, and its expression pattern within the retina has yet to be fully characterized. By contrast, the distribution of POMGNT1 in the neural retina of healthy adult mice, as well as in the 661W cone photoreceptor cell line, has already been described by our group [63]. However, its intracellular distribution and possible colocalization with POMGNT2, which—as exposed above—shares the same substrate with POMGNT1, has not been yet investigated. In the present study, we have analyzed POMGNT2 protein expression in the neural retina of several adult mammalian species, and cultured 661W cells. We then specifically addressed the distribution pattern of POMGNT2 in the retina of healthy monkeys and mice, as well as in the 661W cell line, and compared it with that of POMGNT1 by means of colocalization experiments using confocal microscopy. Notably, in addition to POMGNT2 and POMGNT1 being predominantly located in the ER and the Golgi complex, respectively, both proteins highly accumulated in the nucleus of 661W cells.

## 2. Materials and Methods

*Animals:* The mammalian species analyzed in this study were: mouse (*Mus musculus*, strain C57BL/6J), rat (*Rattus norvegicus*, strain Sprague Dawley), cow (*Bos taurus*) and cynomolgus monkey (*Macaca fascicularis*), including both male and female adult individuals. Each animal was treated as an independent experimental unit for descriptive immunoblotting or immunohistochemistry analyses. Three animals per species were studied (total n = 12). For each animal, the right retina was processed for immunoblotting and the left retina for immunohistochemistry. Compliance with the rules established by the National Institutes of Health (USA), the ARVO Statement for the use of animals in ophthalmic and vision research, and the Directive 2010/63/EU of the European Parliament and Council on the protection of animals used for scientific purposes was a prerequisite for all animal handling. Rodents were housed in the animal-care facility of the Universidad de Alicante under standard laboratory conditions, with food and water ad libitum, controlled temperature and humidity, and a 12 h light/12 h dark photoperiod. Euthanasia was carried out by sequential CO_2_ inhalation and cervical dislocation, and was immediately followed by eye enucleation and storage in RNAlater^®^ solution (Ambion; Austin, TX, USA). The Alicante municipal slaughterhouse supplied the bovine eyes, which were likewise preserved in RNAlater^®^ solution. Monkeys were housed at the animal-care facility of the Universidad de Murcia (Spain) as previously described [78], and their eyes were kindly donated by Dr. M.T. Herrero. All protocols and primate-handling procedures were approved by the university bioethics research committee. Prior to the administration of a lethal overdose of pentobarbital (50 mg/kg, intraperitoneal), young adult monkeys were anesthetized with ketamine (10 mg/kg, i.m.) and their eyeballs were immediately enucleated and stored at −80 °C until protein isolation was carried out from dissected retinas. For immunohistochemistry, mouse and monkey eyes were fixed by immersion in a 4% (*w*/*v*) paraformaldehyde solution in 0.1 M phosphate buffer pH 7.4 (PB) for 1 h, and then subjected to cryoprotection by immersion in sucrose solutions with gradually increasing concentrations [79].

*Cell culture:* The 661W photoreceptor cell line, which provides a reliable and well-characterized in vitro model for studying the inherited and acquired pathogenesis of retinal diseases [80], was kindly provided by Dr. M. Al-Ubaidi (Department of Biomedical Engineering, University of Houston, TX). It was cloned from a retinal tumor of a transgenic mouse line that expressed the artificial *HIT1* transgene, this containing the SV40 large T antigen coding sequence under the control of the human interstitial retinol-binding protein gene (*IRBP*/*RBP3*; Gene ID 5949, OMIM^®^ 180290) promoter [81]. These immortalized cells express cone molecular markers, such as opsins, transducin and cone arrestin, and lack proteins specific to rods, hence constituting a light-sensitive, homogeneous cell line [82,83,84]. The 661W cell line was authenticated using short tandem repeat (STR) genotyping (IDEXX BioResearch, Ludwigsburg, Germany), and their genetic profile was found to be consistent with its expected origin form a mixed FVB × C57BL/6 mouse strain and to carry the *HIT1* transgene that distinguishes this cell line [63,81,85]. The cells were cultured on 75-cm^2^ flasks in high-glucose Dulbecco’s modified Eagle medium (DMEM) from Biowest^®^ (Riverside, MO, USA), containing 10% (*v*/*v*) fetal bovine serum from Capricorn^®^ (Ebsdorfergrund, Germany), 2 mM L-glutamine, and 1% (*v*/*v*) penicillin/streptomycin from Gibco^®^ (Grand Island, NY, USA), at 37 °C in a 5% CO_2_ humidified atmosphere. After 5–10 passages under standard culture conditions, the cells were harvested by enzymatic detachment and centrifugation. the 661W cell pellets obtained were frozen at −80 °C until protein extraction was performed. Alternatively, the cells were re-seeded onto sterile, round glass coverslips, which were placed at the bottom of 24-well culture plates and used for immunocytochemistry once subconfluence was reached.

*Immunoblotting:* As previously described [86], total protein extraction was carried out by adding 20 μL of lysis buffer containing protease inhibitors per 5 mg of retinal tissue or 10^6^ 661W cells. After a 20 min incubation on ice, the extract was clarified by centrifugation at 16,000× *g* at 4 °C for 10 min. The resulting supernatant constituted the total protein fraction. To obtain the cytoplasmic and nuclear protein fractions, the NE-PER kit from Pierce^®^ (Rockford, IL, USA) was used, as previously detailed [87]. Proteins were resolved by sodium dodecyl sulfate-polyacrylamide gel electrophoresis (SDS–PAGE) on 5–20% (*w*/*v*) polyacrylamide-gradient gels, and after electrotransfer to polyvinylidene fluoride (PVDF) membranes from GE Healthcare^®^ (Buckinghamshire, UK), they were stained with SYPRO Ruby Protein Blot Stain from Molecular Probes^®^ (Eugene, OR, USA) to ensure even protein loading and transfer. Immunodetection was performed essentially as described [63,78,86,88]. The membranes were incubated overnight at 4 °C with primary antibody at the dilution indicated in Table 1. This was followed by three 5 min washes with Tris-buffered saline (TBS) pH 7.6, and incubation for 1 h at room temperature with horseradish peroxidase-conjugated anti-IgG secondary antibodies at a 1:10,000 dilution. Finally, development was carried out by using the enhanced chemiluminescence SuperSignal kit from Pierce^®^ followed by exposure of X-ray films (GE Healthcare) to membranes. Cross-contamination between the cytoplasmic and nuclear fractions was discarded by using antibodies against proteins exclusive of these compartments (β-tubulin III and lamin A/C, respectively).

*Immunohistochemistry:* Sixteen μm-thick retinal cryosections were obtained and processed for immunohistochemistry, as previously described [63,79,85,86,88]. Sections were subjected to single or double immunostaining with primary antibodies (Table 1) in PB supplemented with 1% (*v*/*v*) Triton X-100 (PBX) at room temperature overnight. The samples were then incubated at room temperature for 1 h in a humidified, light-protected chamber, in the presence of a 1:100 dilution of donkey secondary antibodies against rabbit or mouse IgG, conjugated to either Alexa Fluor 488 (green) or 546 (red), both from Molecular Probes^®^. To visualize the nuclei, 4’,6-diamidino-2-phenylindole (DAPI) at 10 μg/mL was added simultaneously. Negative-control sections were subjected to the same immunohistochemical procedure as the experimental samples, except that the primary antibody was omitted. Fluorescence was detected using a Zeiss^®^ LSM 800 confocal laser-scanning microscope (Jena, Germany). The JACoP plugin, which was developed for the NIH ImageJ v1.52p software, was used to assess colocalization between coexpressed proteins. The degree of colocalization was evaluated using the Manders colocalization coefficient (MCC), with values near 1 indicating complete colocalization and those near 0 indicating absence of colocalization [89,90]. MCC values were expressed as the mean ± SD from four independently obtained cryosections, in which the retinal layers of interest were cropped using the NIH ImageJ software before analysis. The ranges of colocalization found were designated as follows: very high, 0.8 ≤ MCC ≤ 1.0; high, 0.6 ≤ MCC < 0.8; moderate, 0.4 ≤ MCC < 0.6; low, 0.2 ≤ MCC < 0.4; and not detectable, 0 ≤ MCC < 0.2 [91].

*Immunocytochemistry:* A total of 75,000 cells per well were seeded onto 24-well plates and cultured until reaching near confluence. Subsequently, 661W cells were fixed with 4% (*w*/*v*) paraformaldehyde dissolved in Dulbecco’s PBS (DPBS), washed three times with DPBS, and permeabilized with 0.2% (*v*/*v*) Triton X-100 diluted in DPBS for 10 min at each step. To block non-specific antibody binding, samples were incubated for 1 h with 1% (*w*/*v*) bovine serum albumin (BSA) in DPBS. Primary antibodies, diluted in blocking solution as specified in Table 1, were applied overnight at 4 °C in a humidified chamber. Subsequent incubation with Alexa Fluor-conjugated secondary antibodies, processing of negative controls and fluorescence imaging were performed as described in the previous section. MCC values were expressed as the mean ± SD from 10 cells analyzed from micrographs obtained in duplicate experiments, in which the cells of interest were cropped using the NIH ImageJ software before analysis.

## 3. Results

### 3.1. Expression of POMGNT2 in the Mammalian Retina and the 661W Photoreceptor Cell Line

**Figure 1:**
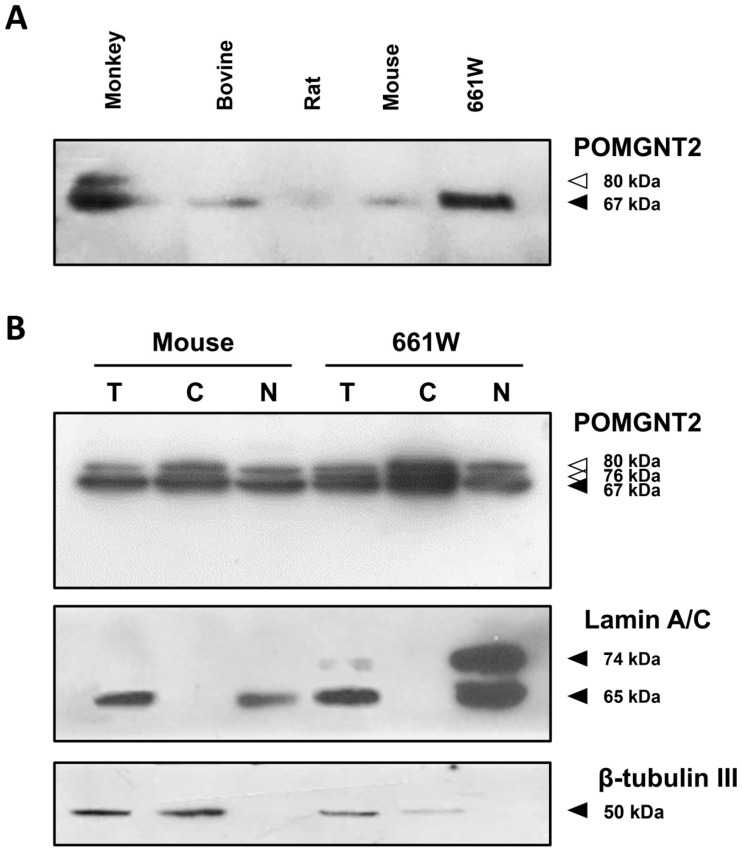
Expression of POMGNT2 protein in mammalian retinas and the 661W photoreceptor cell line. (**A**) Immunoblotting analysis of POMGNT2 on total protein extracts (100 μg/lane) from neural retinas of the indicated species and 661W cells. (**B**) Immunoblotting analysis of POMGNT2, β-tubulin III, and lamin A/C on total (T), cytoplasmic (C) and nuclear (N) fractions (50 μg of protein/lane) extracted from the mouse neural retina and 661W cells. Apparent molecular masses are shown on the right side of each panel (solid arrowheads, canonical proteins; open arrowheads, POMGNT2 isoforms).

The expression profile of the DGP-associated enzyme POMGNT2 remains unexplored in the context of the mammalian retina. In order to assess whether this protein is present in this tissue, total protein was isolated from neural retinas collected from different species. We found by immunoblotting that this protein was actually synthesized in the retina and 661W cells. As illustrated in Figure 1A, POMGNT2 was present in retinal extracts from all the species analyzed, as well as in cultured 661W photoreceptors, exhibiting an apparent molecular mass of 67 kDa consistent with its predicted size based on the amino acid sequence. Additionally, we detected a POMGNT2 enzyme form migrating with an apparent molecular mass of 80 kDa in the monkey retina (Figure 1A). We next examined the intracellular localization of POMGNT2 using cytoplasmic and nuclear protein extracts from the mouse neural retina and the 661W cell line. As shown in Figure 1B, POMGNT2 was present in both cytoplasmic and nuclear fractions, migrating with apparent molecular masses of 67 and 80 kDa in all protein extracts analyzed. In addition, a third band of 76 kDa was noticeable in the cytoplasmic fraction of the mouse retina, at a lower level than the 80 and 67 kDa bands. The appearance of the 76 and 80 kDa bands, in addition to the canonical 67 kDa enzyme form, was likely attributable to post-translational modifications of the POMGNT2 protein, such as *O*- and *N*-glycosylation, ubiquitination and/or phosphorylation, as detected in other studies [92,93,94], or predicted by the Net*O*Glyc (v4.0), Net*N*Glyc (v1.0) and PhosphoSitePlus (v6.8.1) software. The absence of non-specific bands of other sizes supports the reliability of the antibody to POMGNT2 used. Using antibodies against β-tubulin III and lamins A and C, which are traditionally considered cytoplasmic- and nuclear-specific markers, respectively, we confirmed that our fractionation procedure did not lead to cross-contamination between the two subcellular fractions obtained from the mouse retina and 661W cells (Figure 1B).

### 3.2. Immunolocalization of POMGNT2 and POMGNT1 in the Monkey and Mouse Retinas

**Figure 2:**
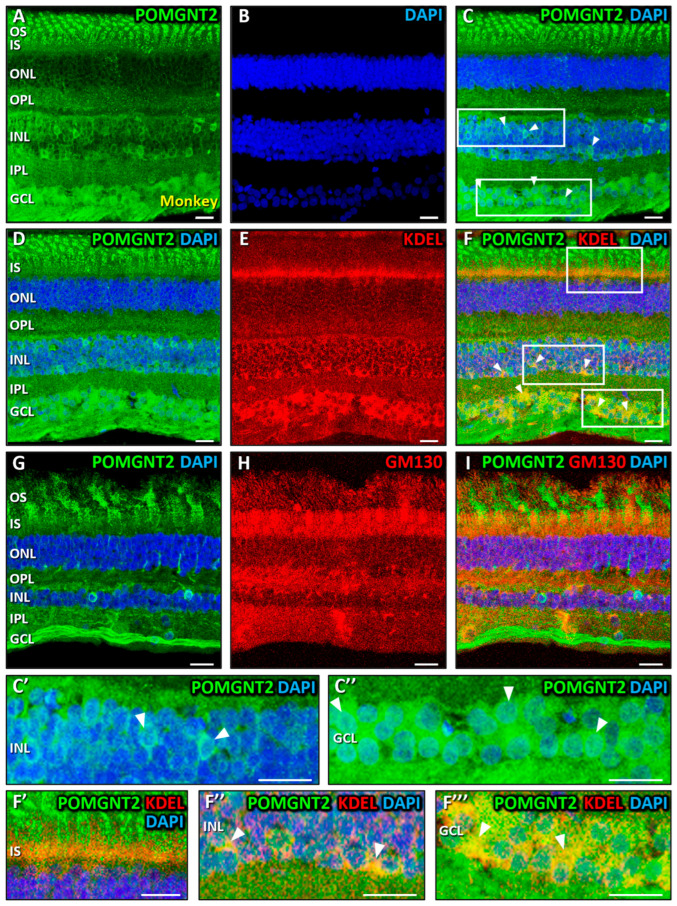
Immunolocalization of POMGNT2 in the monkey retina. Double immunostaining was carried out on monkey retinal sections for POMGNT2 (green; (**A**,**C**,**D**,**F**,**G**,**I**)), and the specific ER marker KDEL (red; (**E**,**F**)) or the specific Golgi complex marker GM130 (red; (**H**,**I**)). Enlarged views in (**C’**,**C’’**) correspond to boxed areas in (**C**), where white arrowheads point to colocalization of POMGNT2 with DAPI (in cyan), while (**F’**,**F’’**,**F’’’**) correspond to boxed areas in (**F**), while arrowheads point to colocalization of POMGNT2 with KDEL (in yellow). Nuclei were counterstained with DAPI (blue; (**B**,**C**,**D**,**F**,**G**,**I**)). POMGNT2 immunoreactivity was detected in both the cytoplasm and nuclei of cells in the INL and GCL of the monkey retina, colocalizing with DAPI ((**C**), arrowheads). Colocalization with KDEL was observed in the IS of photoreceptors (**F’**) and in the INL and GCL ((**D**–**F**,**F’’**,**F’’’**); arrowheads), whereas no significant colocalization with GM130 was detected (**I**). Abbreviations: OS, outer segments; IS, inner segments; ONL, outer nuclear layer; OPL, outer plexiform layer; INL, inner nuclear layer; IPL, inner plexiform layer; GCL, ganglion cell layer. Each bar equals 20 μm.

Next, we aimed to examine the spatial distribution of POMGNT2 in the retina of adult monkeys and mice using confocal immunofluorescence microscopy. In monkey retinal cryosections POMGNT2 displayed a widespread distribution across layers, but was predominantly localized in the inner (IS) and outer (OS) segments of photoreceptors (Figure 2A,C). Additionally, POMGNT2 was detected in the inner nuclear (INL) and ganglion cell (GCL) layers, moderately colocalizing with the DNA-binding dye, DAPI, in some nuclei residing in the INL (Figure 2C,C’, arrowheads; MCC = 0.52 ± 0.09) and in all nuclei of cells in the GCL (Figure 2C,C’’, arrowheads; MCC = 0.56 ± 0.07). We also observed a weaker immunoreactivity of POMGNT2 in the outer plexiform layer (OPL), where it concentrated in the synaptic terminals of photoreceptors, and in the inner plexiform layer (IPL) (Figure 2A,C). In order to discern whether cytoplasmic POMGNT2 in retinal cells pertained to the ER and/or the Golgi complex, we first performed a double immunostaining for POMGNT2 (Figure 2D) and KDEL (Figure 2E). This is a signal sequence present in numerous proteins located in the lumen of the ER that prevents them from being secreted into the extracellular medium [95] and which is commonly used as a marker for this organelle. As shown, a moderate colocalization of the immunolabelings for POMGNT2 and KDEL was found in the IS of photoreceptors (Figure 2F,F’, yellow; MCC = 0.52 ± 0.06), as well as in the INL (Figure 2F,F’’, arrowheads; MCC = 0.56 ± 0.03), and the GCL (Figure 2F,F’’’, arrowheads; MCC = 0.56 ± 0.06), this indicating that POMGNT2 was located in the ER of photoreceptors, cells residing in the INL and ganglion cells in the monkey retina. Likewise, a double immunostaining was performed using antibodies against POMGNT2 (Figure 2G) and GM130 (Figure 2H), a structural membrane protein of the cis-Golgi that helps preserving Golgi architecture and promotes vesicle fusion to the Golgi membrane [96], serving as a well-established marker of this organelle. In this case, no significant colocalization was detected, however, between POMGNT2 and the Golgi marker in any of the four layers in which POMGNT2 was expressed (Figure 2I), namely the photoreceptors IS (MCC = 0.18 ± 0.04), the OPL (MCC = 0.06 ± 0.07), the INL (MCC = 0.11 ± 0.06) and the GCL (MCC = 0.18 ± 0.05), which was indicative that POMGNT2 was not localized in this organelle in the monkey retina.

**Figure 3:**
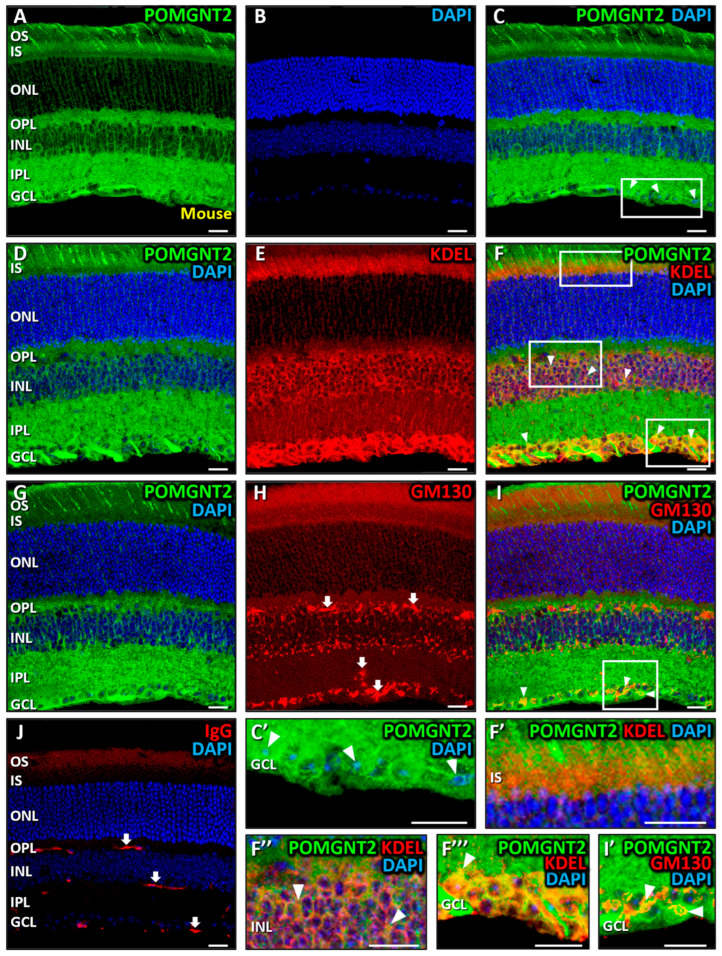
Immunolocalization of POMGNT2 in the mouse retina. Double immunostaining was carried out on mouse retinal sections for POMGNT2 (green; (**A**,**C**,**D**,**F**,**G**,**I**)) and the specific ER marker KDEL (red; (**E**,**F**)), or the specific Golgi complex marker GM130 (red; (**H**,**I**)). The enlarged view in (**C’**) corresponds to the boxed area in (**C**), the views in (**F’**,**F’’**,**F’’’**) to boxed areas in (**F**), and the view in (**I’**) to the boxed area in (**I**). Nuclei were counterstained with DAPI (blue; (**B**,**C**,**D**,**F**,**G**,**I**,**J**)). In the mouse retina, POMGNT2 immunoreactivity was detected in both the cytoplasm and nuclei of GCL cells, colocalizing with DAPI ((**C**,**C’**), arrowheads). Colocalization with KDEL (in yellow) was observed in the IS of photoreceptors ((**F**,**F’**), arrowheads), and in the INL and GCL ((**F**,**F’’**,**F’’’**), arrowheads), while colocalization with GM130 (in yellow) was detected in the GCL ((**I**,**I’**), arrowheads). Arrows in (**H**,**J**) indicate non-specific labeling of retinal vessels by secondary antibodies to mouse IgG. Retinal layer abbreviations are spelled out in the legend of Figure 2. Each bar equals 20 μm.

We next investigated the distribution profile of POMGNT2 in cryostat sections of the mouse retina. Similar to our observations in monkeys, POMGNT2 was detected across multiple retinal layers, showing an enrichment in the inner (IS) and outer (OS) segments of photoreceptors (Figure 3A,C). Additionally, POMGNT2 was present in the OPL, where it was concentrated in synaptic terminals, as observed in the monkey retina, and in the IPL. Furthermore, POMGNT2 was located in the INL, although without colocalizing with DAPI (Figure 3C; MCC = 0.03 ± 0.02) at variance with the monkey retina, and in the GCL, where it moderately colocalized with DAPI in all cell nuclei (Figure 3C,C’, arrowheads; MCC = 0.56 ± 0.12). These findings confirmed that POMGNT2 was present not only in the cytoplasm but also in the nucleus in a variety of retinal cell types, in agreement with our immunoblotting results (Figure 1B). In order to assess whether POMGNT2 was located in the ER of mouse retinal cells, double immunostaining was carried out with antibodies against POMGNT2 (Figure 3D) and KDEL (Figure 3E). The results obtained revealed a high colocalization of the immunolabeling for both polypeptides in the IS of photoreceptors (Figure 3F,F’, yellow; MCC = 0.61 ± 0.06), as well as in the INL (Figure 3F,F’’, arrowheads; MCC = 0.74 ± 0.04), and a very high colocalization in the GCL (Figure 3F,F’’’, arrowheads; MCC = 0.86 ± 0.10). These results indicated that POMGNT2 was present in the ER of photoreceptors, INL cells and ganglion cells in the mouse retina, consistent with our observations in the monkey (see above). Subsequently, we carried out a double immunostaining with antibodies to POMGNT2 (Figure 3G) and GM130 (Figure 3H). In this case, the signal that we detected from retinal vessels in the OPL, IPL and GCL was non-specific (Figure 3H, arrows), since it was also obtained in the absence of the primary antibody (Figure 3J, arrows). As also shown, no colocalization was seen between POMGNT2 and GM130 (Figure 3I) in the IS of photoreceptors (MCC = 0.14 ± 0.08), in the OPL (MCC = 0.11 ± 0.09) or in cells residing in the INL (MCC = 0.14 ± 0.12). However, we detected a very high colocalization between POMGNT2 and the Golgi marker in the GCL (Figure 3I,I’, arrowheads; MCC = 0.81 ± 0.14), this indicating the presence of the POMGNT2 protein, in addition to the ER, in the Golgi of ganglion cells in the mouse retina, in contrast to our observations in the monkey retina (see above).

**Figure 4:**
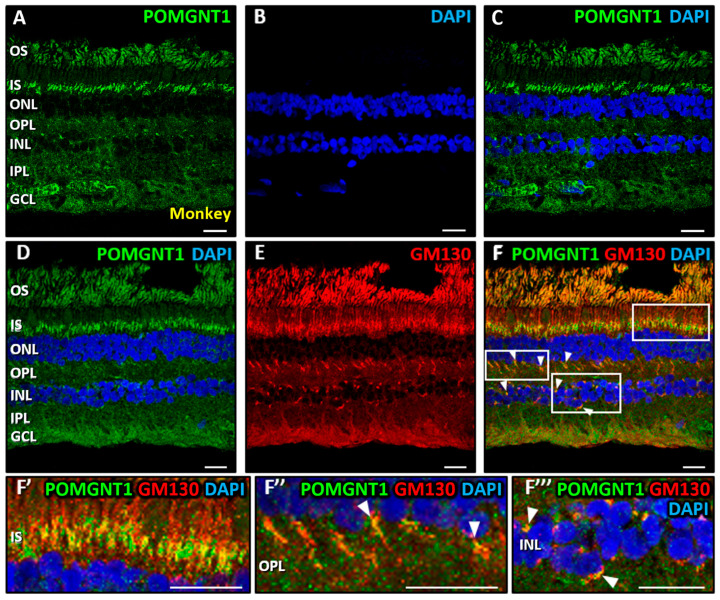
Immunolocalization of POMGNT1 in the monkey retina. Double immunostaining was carried out on monkey retinal sections for POMGNT1 (green; (**A**,**C**,**D**,**F**)) and the specific Golgi complex marker GM130 (red; (**E**,**F**)). Enlarged views in (**F’**,**F’’**,**F’’’**) correspond to boxed areas in (**F**). Nuclei were counterstained with DAPI (blue; (**B**,**C**,**D**,**F**)). POMGNT1 immunoreactivity was not found in any cell nuclei in the monkey retina. However, colocalization of POMGNT1 with GM130 (in yellow) was observed in the IS of photoreceptors (**F**,**F’**), in the OPL and in cells of the INL (**F**,**F’’**,**F’’’**) (arrowheads). Abbreviations of retinal layers are spelled out in the legend of Figure 2. Each bar equals 20 μm.

Previous experiments conducted in our laboratory demonstrated that, in the mouse retina, POMGNT1 was exclusively present in the photoreceptor IS layer [63]. Therefore, in the present work we focused on studying the distribution pattern of POMGNT1 and its subcellular localization in the monkey retina. In monkey retinal sections immunostained with a POMGNT1-specific antibody, we observed that this protein was present across multiple retinal layers (Figure 4A,C), although with predominant accumulation in the photoreceptor IS and, to a lesser extent, in the OS, OPL, INL and GCL. However, no significant colocalization was detected between POMGNT1 and DAPI in any of the two inner retinal nuclear layers (Figure 4C), i.e., the INL (MCC = 0.06 ± 0.03) and the GCL (MCC = 0. 09 ± 0.04). In double immunostainings with POMGNT1 (Figure 4D) and GM130 (Figure 4E), we observed a moderate colocalization in all layers where POMGNT1 was present, i.e., the photoreceptor IS (Figure 4F,F’, yellow; MCC = 0.58 ± 0.04) and the OPL, where photoreceptor axon terminals are located (Figure 4F,F’’, arrowheads; MCC = 0.59 ± 0.05), with additional colocalization in some cells in the INL (Figure 4F,F’’’, arrowheads; MCC = 0.75 ± 0.11). These results indicated that the POMGNT1 protein was present in the Golgi complex of photoreceptors, in consonance with the results obtained by our group in the mouse retina [63], but additionally in cells residing in the INL in the monkey retina

**Figure 5:**
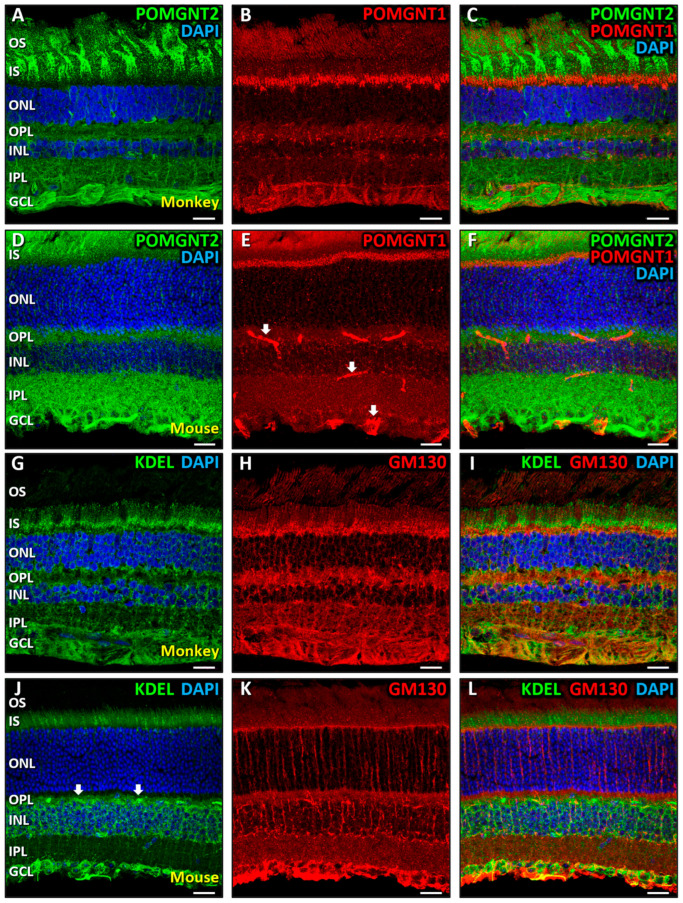
Coimmunolocalization of POMGNT2 and POMGNT1 proteins with KDEL and GM130 markers, respectively, in the monkey and mouse retinas. Double immunolabelings were conducted on monkey retinal sections for POMGNT2 (green; (**A**,**C**)) and POMGNT1 (red; (**B**,**C**)), as well as on mouse retinal sections for POMGNT2 (green; (**D**,**F**)) and POMGNT1 (red; (**E**,**F**)). Also, double immunostainings were performed on monkey retinal sections for the specific ER marker KDEL (green; (**G**,**I**)) and the specific Golgi complex marker GM130 (red; (**H**,**I**)), as well as on mouse retinal sections for KDEL (green; (**J**,**L**)) and GM130 (red; (**K**,**L**)). Nuclei were counterstained with DAPI (blue; (**A**,**C**,**D**,**F**,**G**,**I**,**J**,**L**)). No colocalization of POMGNT2 with POMGNT1 (**C**,**F**) or of KDEL with GM130 (**I**,**L**) was observed in any retinal layer of the two species studied. Arrows in (**E**,**J**) indicate non-specific labeling of retinal vessels by secondary antibodies to mouse IgG. Retinal layer abbreviations are spelled out in the legend of Figure 2. Each bar equals 20 μm.

Once the pattern of distribution and subcellular localization of POMGNT2 and POMGNT1 in the retinas of adult monkey and mouse had been analyzed, we proceeded to jointly study the expression of both proteins in the retinas of the two species, given that they are enzymes that share the same substrate but should carry out their function in α-DG glycosylation in an appropriate order and intracellular sites [40]. Toward this end, a double immunostaining was first performed using antibodies against POMGNT2 (Figure 5A) and POMGNT1 (Figure 5B) on monkey retinal sections. Consistently with our previous results (Figure 2), POMGNT2 was observed to distribute between the OS and IS of monkey photoreceptors, as well as in the two plexiform layers (OPL and IPL) and the two nuclear layers of the inner retina (INL and GCL) (Figure 5C). Analogously, POMGNT1 was located in the IS of photoreceptors, OPL, INL and GCL (Figure 5B). However, no significant colocalization was detected in this assay between POMGNT2 and POMGNT1 proteins in any of the layers of the monkey retina, where both proteins were coexpressed (Figure 5C), namely the photoreceptors IS (MCC = 0.14 ± 0.01), the OPL (MCC = 0.16 ± 0.02), the INL (MCC = 0.16 ± 0.05) or the GCL (MCC = 0.18 ± 0.01). Next, double immunolabeling experiments were performed using antibodies against POMGNT2 (Figure 5D) and POMGNT1 (Figure 5E) in mouse cryostat retinal sections. In coherence with our previous results (Figure 3), POMGNT2 was present both in the OS and IS of photoreceptors, as well as in the OPL, IPL, INL and GCL. However, POMGNT1 was visibly present only in the IS of photoreceptors, as previously defined [63]. No significant colocalization was detected between these proteins in the IS of photoreceptors (Figure 5F; MCC = 0.14 ± 0.03), the only layer of the mouse retina where both were expressed. These results indicated that POMGNT2 and POMGNT1 were located at the subcellular level in different areas in both the monkey and mouse retinas. In order to ascertain whether the intracellular distribution of the ER and the Golgi complex coincided with that of POMGNT2 and POMGNT1, respectively, we performed a double immunolabeling using antibodies against markers of these two organelles, i.e., KDEL (ER) and GM130 (Golgi complex) in monkey (Figure 5G,H) and mouse (Figure 5J,K) retinal sections. As shown, in both mammals, the ER was observed to be contained in the IS of photoreceptors, INL and GCL (Figure 5G,I,J,L), while the Golgi complex was located in the IS of photoreceptors, OPL, INL and GCL (Figure 5H,I,K,L). As expected, no significant colocalization between KDEL and GM130 was detected in any of the retinal layers in the two species, i.e., the IS of photoreceptors (Figure 5I; MCC = 0.14 ± 0.04; Figure 5L; MCC = 0.15 ± 0.03), the INL (Figure 5I; MCC = 0.09 ± 0.03; Figure 5L; MCC = 0.13 ± 0.03) or the GCL (Figure 5I; MCC = 0.14 ± 0.06; Figure 5L; MCC = 0.13 ± 0.04). These results indicated that the distribution patterns of POMGNT2 and POMGNT1 proteins were coherently compatible with those of the ER and the Golgi complex, respectively, in the retinas of the two species studied, but were not coexpressed in any of these two organelles.

### 3.3. Immunolocalization of POMGNT2 and POMGNT1 in the 661W Photoreceptor Cell Line

**Figure 6:**
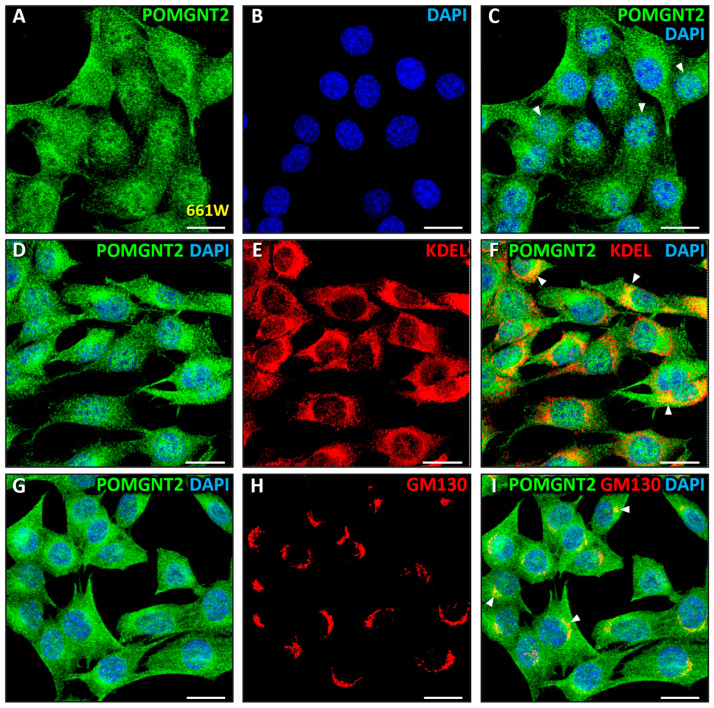
Immunolocalization of POMGNT2 in cultured photoreceptors of the 661W cell line. Cells were immunostained with specific antibodies to POMGNT2 (green; (**A**,**C**,**D**,**F**,**G**,**I**)), and the ER marker KDEL (red; (**E**,**F**)) or the Golgi marker GM130 (red; (**H**,**I**)). Nuclei were counterstained with DAPI (blue; (**B**,**C**,**D**,**F**,**G**,**I**)). POMGNT2 was present in both the cytoplasm and nuclei of 661W cells, showing nuclear colocalization with DAPI ((**C**), arrowheads), and cytoplasmic colocalization (in yellow) with both KDEL ((**F**), arrowheads) and GM130 ((**I**), arrowheads). Each bar equals 20 μm.

We then set out to investigate whether the POMGNT2 intracellular distribution pattern seen in the monkey and mouse photoreceptors was extrapolatable to the 661W photoreceptor cell line. Figure 6A,C shows that in these cells POMGNT2 was detected in the cytoplasm as well as in the nucleus, consistently with our immunoblotting results (Figure 1B). POMGNT2 exhibited a dense immunoreactivity in the nuclei, displaying a punctate pattern, yet showing a moderate colocalization with DAPI (Figure 6C, arrowheads; MCC = 0.59 ± 0.08). This protein was also broadly distributed in the cytoplasm of 661W cells and, in analogy to retinal sections, POMGNT2 displayed a location that was compatible with that of the ER. In order to further investigate this possibility, we performed a double immunostaining using antibodies specific to POMGNT2 (Figure 6D) and KDEL (Figure 6E). As a result, we detected a high colocalization between POMGNT2 and the ER marker (Figure 6F, arrowheads; MCC = 0.69 ± 0.10), this indicating that POMGNT2 was enriched in the ER in the 661W cell line. In addition, we performed a double immunolabeling using antibodies against POMGNT2 (Figure 6G) and GM130 (Figure 6H). As a result of this analysis, a very high colocalization was detected between POMGNT2 and the Golgi complex marker (Figure 6I, arrowheads; MCC = 0.95 ± 0.06). These results indicated that POMGNT2 was located in the Golgi complex, in addition to the ER, in the 661W photoreceptor cell line, while in photoreceptors of the monkey and mouse retinas POMGNT2 was located exclusively to the ER, as shown in Figure 1 and Figure 2.

**Figure 7:**
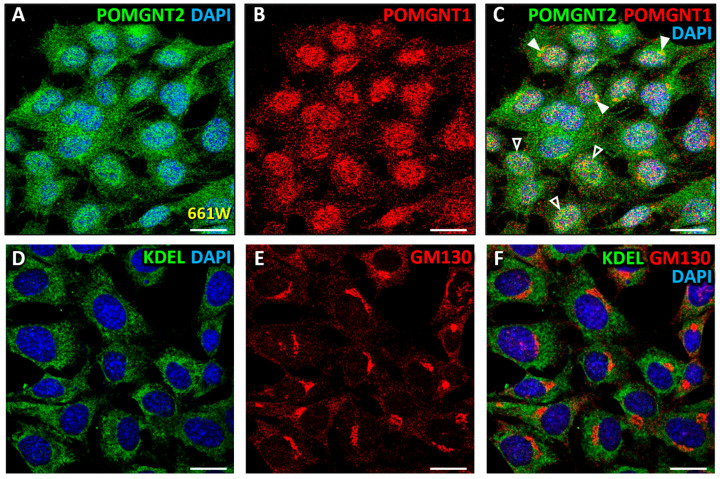
Coimmunolocalization of POMGNT2 and POMGNT1 proteins with KDEL and GM130 markers, respectively, in 661W photoreceptors. Cells were doubly immunostained with antibodies specific for POMGNT2 (green; (**A**,**C**)) and POMGNT1 (red; (**B**,**C**)). Also, double immunostainings were performed for the specific ER marker KDEL (green: **D**,**F**) and the specific Golgi complex marker GM130 (red; (**E**,**F**)). Nuclei were counterstained with DAPI (blue; (**A**,**C**,**D**,**F**)). POMGNT2 and POMGNT1 were observed to colocalize (in yellow) in the cytoplasm and the nucleus of 661W photoreceptors ((**C**), closed and open arrowheads, respectively), but no colocalization of KDEL with GM130 was observed in this cell line (**F**). Each bar equals 20 μm.

Since POMGNT1 had been previously located by our group in both the Golgi complex and the nucleus in 661W photoreceptors [63], we decided to carry out a double immunostaining for POMGNT2 (Figure 7A) and POMGNT1 (Figure 7B) in this cell line. As a result of this assay, high colocalization was detected between both proteins in a discrete region of the cytoplasm (Figure 7C, closed arrowheads; MCC = 0.63 ± 0.05) that corresponded in its appearance to the Golgi complex, this corroborating our previous results pointing out a localization of POMGNT2 and POMGNT1 in this organelle. Additionally, a high colocalization was detected between the two proteins inside the nuclei of 661W cells (Figure 7C, closed arrowheads; MCC = 0.60 ± 0.08). In the same way as we had previously carried out with the mammalian retinas studied, we performed a double immunolabeling using antibodies against the ER and Golgi complex markers, KDEL and GM130, respectively, in 661W photoreceptors (Figure 7D,E) to ascertain whether the intracellular distribution of these organelles coincided with that of POMGNT2 and POMGNT1, respectively. The ER was observed to be widely spread throughout the cytoplasm (Figure 7D,F), while the Golgi complex was located in a discrete region of the cytoplasm next to the nucleus (Figure 7E,F). As expected, no significant colocalization between KDEL and GM130 was detected in the cytoplasm (Figure 7F; MCC = 0.12 ± 0.04). These results indicated that the distribution patterns of the ER and Golgi complex in the cytoplasm of 661W photoreceptors were coherently compatible with POMGNT2 and POMGNT1 proteins colocalizing in the Golgi complex in addition to the nucleus.

### 3.4. Intranuclear Distribution of POMGNT2 and POMGNT1 in the 661W Photoreceptor Cell Line

**Figure 8:**
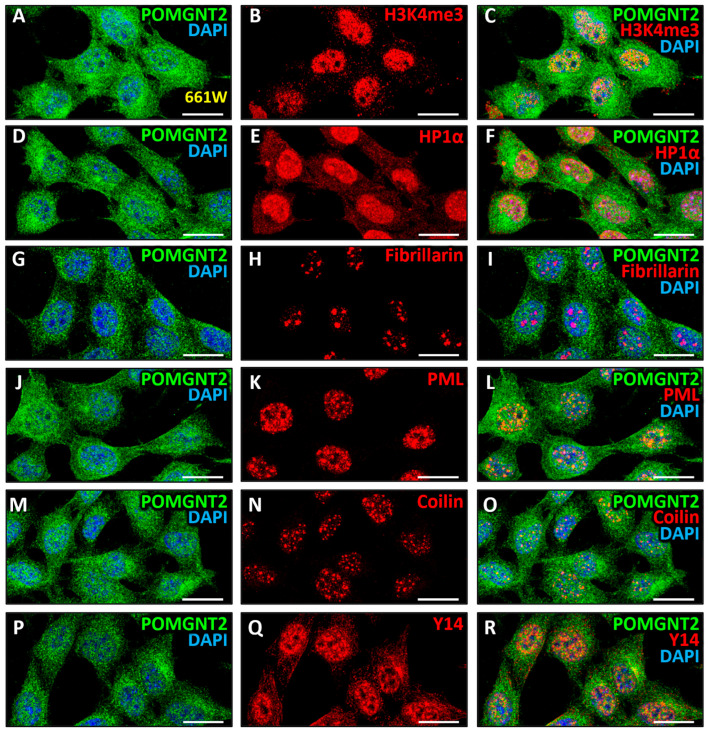
Intranuclear distribution of POMGNT2 in the photoreceptor cell line 661W. Cells were doubly immunostained for both POMGNT2 (green; (**A**,**C**,**D**,**F**,**G**,**I**,**J**,**L**,**M**,**O**,**P**,**R**)) and either the euchromatin marker H3K4me3 (red; (**B**,**C**)), the heterochromatin marker HP1α (red; (**E**,**F**)), the nucleoli marker fibrillarin (red; (**H**,**I**)), the promyelocytic-leukemia nuclear body marker PML (red; (**K**,**L**)), the Cajal nuclear body marker coilin (red; (**N**,**O**)), or the spliceosome marker Y14 (red; (**Q**,**R**)). Nuclei were counterstained with DAPI (blue; (**A**,**C**,**D**,**F**,**G**,**I**,**J**,**L**,**M**,**O**,**P**,**R**)). POMGNT2 immunoreactivity was detected in the nucleus of 661W cells, with colocalization being observed (in yellow) with H3K4me3 (**C**), HP1α (**F**), PML (**L**), coilin (**O**) and Y14 (**R**). No colocalization of POMGNT2 with fibrillarin, however, was detected (**I**). Each bar equals 20 μm.

Lastly, having confirmed the presence of POMGNT2 and POMGNT1 proteins, as well as their colocalization, in the nucleus of 661W cells (Figure 1B, Figure 6A,C and Figure 7A,C), where the localization and function of these proteins have not been previously described, we then set out to investigate in more depth the intranuclear localization of these proteins. First, a study of the possible localization of POMGNT2 in chromatin (euchromatin and heterochromatin) was performed. With this purpose, we carried out a double immunostaining for POMGNT2 (Figure 8A) and the euchromatin marker H3K4me3 (Figure 8B), i.e., histone H3 trimethylated at amino acid Lys-4, which is associated with sites of active gene expression [97]. In this assay, we observed a moderate colocalization between both proteins (Figure 8C; MCC = 0.52 ± 0.06) in the nucleus of 661W cells, this indicating that POMGNT2 was associated with euchromatin in this cell line. Next, a double immunolabeling was performed using specific antibodies to POMGNT2 (Figure 8D) and the heterochromatin marker HP1α (Figure 8E), which binds to histone H3 trimethylated at amino acid Lys-9 (H3K9me3) to regulate the formation, function and structure of heterochromatin [98]. As a result, we detected a moderate colocalization between POMGNT2 and HP1α (Figure 8F; MCC = 0.46 ± 0.04), indicative that POMGNT2 was also associated with heterochromatin in 661W cells. Thereafter, the localization of POMGNT2 in particular nucleoplasmic structures was analyzed, in order to determine the presence of this protein in different functional compartments of the nucleus, and hence to address its possible function within this organelle. The first of such structures in which the possible location of POMGNT2 was studied was the nucleolus, an organelle known to be involved in multiple nuclear functions, such as rRNA transcription, ribosome biogenesis, mitosis regulation, cell cycle progression and proliferation, and the cellular response to stress [99]. With this purpose, a double immunostaining was performed using specific antibodies to POMGNT2 (Figure 8G) and the nucleolus marker fibrillarin (Figure 8H), a methyltransferase located exclusively in the fibrillar region of the nucleolus where it carries out the methylation of rRNA and histone H2A [100,101,102]. As a result of this experiment, no significant colocalization was detected between POMGNT2 and fibrillarin (Figure 8I; MCC = 0.14 ± 0.05), indicating that POMGNT2 was not localized in the nucleoli of 661W cells.

Next, we analyzed the localization of POMGNT2 in promyelocytic leukemia nuclear bodies (PML), which are involved in a wide variety of relevant cellular processes, including transcriptional and cell division regulation, tumor suppression, apoptosis, senescence, DNA damage response and genome stability, as well as viral defense [103]. Therefore, a double immunolabeling was carried out using specific antibodies against POMGNT2 (Figure 8J) and the PML protein (Figure 8K), the main component of such nuclear bodies which is also essential for their formation [104]. This assay showed a moderate colocalization between both proteins (Figure 8L; MCC = 0.55 ± 0.06), which suggested that POMGNT2 was a constituent protein of PML nuclear bodies. The presence of this protein in Cajal nuclear bodies, which are involved in the synthesis of small nuclear and nucleolar ribonucleoproteins (snRNPs and snoRNPs, respectively), both of which are required for RNA transcription and processing [105], was also studied. A double immunostaining was carried out using specific antibodies against POMGNT2 (Figure 8M) and the marker of Cajal nuclear bodies called coilin (Figure 8N), a nuclear phosphoprotein that accumulates only in these structures and is essential for their correct formation [106]. These results revealed a moderate colocalization between POMGNT2 and coilin proteins (Figure 8O; MCC = 0.54 ± 0.10), indicating that POMGNT2 was concentrated in Cajal nuclear bodies. Next, the possible location of POMGNT2 in spliceosomes of 661W cells was analyzed. These are organelles of 4.8 MDa forming part of nuclear speckles constituted by ribonucleoproteins that carry out mRNA splicing reactions [107,108]. With this purpose, an immunostaining was carried out using specific antibodies to POMGNT2 (Figure 8P) and the spliceosome marker Y14 (Figure 8Q). This is an RNA-binding protein that constitutes the main component of the multiprotein exon junction complex, which is part of the spliceosome and is associated with splicing sites in the mRNA [109]. As a result of this assay, a moderate colocalization was obtained between both proteins (Figure 8R; MCC = 0.45 ± 0.06), suggesting that POMGNT2 was also partially concentrated in nuclear speckles of 661W cells.

**Figure 9:**
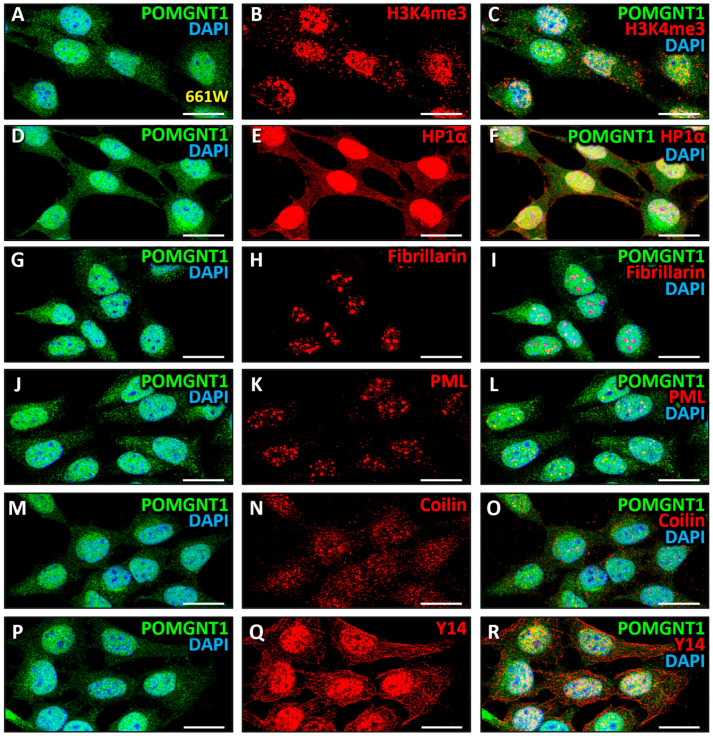
Intranuclear distribution of POMGNT1 in the photoreceptor cell line 661W. Cells were doubly immunostained for both POMGNT1 (green; (**A**,**C**,**D**,**F**,**G**,**I**,**J**,**L**,**M**,**O**,**P**,**R**)) and either the euchromatin marker H3K4me3 (red; (**B**,**C**)), the heterochromatin marker HP1α (red; (**E**,**F**)), the nucleoli marker fibrillarin (**H**,**I**), the marker of promyelocytic-leukemia nuclear bodies PML (red; (**K**,**L**)), the Cajal nuclear body marker coilin (red; (**N**,**O**)), or the spliceosome marker Y14 (red; (**Q**,**R**)). Nuclei were counterstained with DAPI (blue; (**A**,**C**,**D**,**F**,**G**,**I**,**J**,**L**,**M**,**O**,**P**,**R**)). POMGNT1 immunoreactivity was present in the nucleus of 661W cells, with colocalization being observed (in yellow) with H3K4me3 (**C**), HP1α (**F**), PML (**L**), coilin (**O**) and Y14 (**R**). No colocalization of POMGNT2 with fibrillarin, however, was observed (**I**). Each bar equals 20 μm.

Finally, we analyzed in more depth the subnuclear localization of POMGNT1 in the 661W photoreceptor cell line, following the same methodology we had previously used for POMGNT2. First, a study of the possible localization of POMGNT1 in chromatin (euchromatin and/or heterochromatin) was performed. Toward this end, a double immunolabeling was carried out using specific antibodies to POMGNT1 (Figure 9A), in this case a new antibody not previously used in our laboratory, and the euchromatin marker H3K4me3 (Figure 9B). The results of this experiment showed a high colocalization between POMGNT1 and this marker (Figure 9C; MCC = 0.78 ± 0.06), indicating that POMGNT1 was localized in euchromatin in 661W cells. A double immunostaining was then performed using specific antibodies against POMGNT1 (Figure 9D) and the heterochromatin marker HP1α (Figure 9E). This assay revealed a very high colocalization between the POMGNT1 enzyme and HP1α (Figure 9F; MCC = 0.86 ± 0.04), indicative that POMGNT1 was also localized in heterochromatin. Second, the presumptive localization of POMGNT1 in other nucleoplasmic structures was analyzed, in order to investigate the presence of this protein in different functional compartments of the nucleus, and thus to address its possible function in this organelle. The first nucleoplasmic structure in which the possible location of POMGNT1 was studied was the nucleolus, by performing a double immunolabeling with specific antibodies against POMGNT1 (Figure 9G) and the nucleolus marker fibrillarin (Figure 9H). As a result of this experiment, no significant colocalization was detected between both proteins (Figure 9I; MCC = 0.16 ± 0.08), suggesting that POMGNT1 was excluded from the nucleolus.

Then, we decided to study the possible localization of POMGNT1 in PML nuclear bodies, by carrying out a double immunolabeling using specific antibodies against POMGNT1 (Figure 9J) and PML (Figure 9K). The results of this assay revealed a very high colocalization between both proteins (Figure 9L; MCC = 0.89 ± 0.07), suggesting that POMGNT1 was also present in PML nuclear bodies. Additionally, the presence of POMGNT1 in Cajal nuclear bodies was analyzed by performing a double immunostaining using specific antibodies against POMGNT1 (Figure 9M) and the marker of such structures called coilin (Figure 9N). As a result of this experiment, a high colocalization was obtained between both proteins (Figure 9O; MCC = 0.75 ± 0.09), indicating that POMGNT1 was also located in Cajal nuclear bodies. To finish, the localization of POMGNT1 in nuclear speckles was assessed by carrying out a double immunostaining with specific antibodies against POMGNT1 (Figure 9P) and the spliceosome marker, Y14 (Figure 9Q). The results of this assay evidenced a very high colocalization between both proteins (Figure 9R; MCC = 0.87 ± 0.05), this being indicative that POMGNT1 protein was also present in spliceosomes, and hence in nuclear speckles of 661W cells.

## 4. Discussion

### 4.1. Intracellular Distribution of Dystroglycanopathy-Associated Proteins in Mammalian Retinal Cells

To date, retinal expression of particular DGP-associated genes at the mRNA level has been revealed through in situ hybridization, namely of *Pomt1* in the mouse embryonic retina [110] and *FKTN* in the human fetal retina [36]. At the protein level, our group has previously reported expression of POMT1 in the adult mouse retina [111], as well as of POMGNT1 [63], fukutin/FKTN and fukutin-related protein (FKRP) [85]. Given that little is known about DGP-associated genes and proteins in the mammalian retina, our research has focused on the expression in this tissue of the six best-known genes, namely *POMT1*, *POMT2*, *POMGNT1*, *FKTN*, *FKRP* and *LARGE1*, whose encoded proteins act at the very first or last steps of the α-DG *O*-mannosyl glycosylation pathway. We aimed thereby at shedding light on the function in the retina of the proteins encoded by these genes, and hence to clarify how their absence or dysfunction contributes to the ocular defects that DGPs entail. In this fashion, it was determined that all of them were expressed at both mRNA and protein levels in the neural retina of adult monkeys, cows, rats and mice, as well as in the mouse 661W photoreceptor cell line ([63,85,111]; Quereda C, Uribe ML, Martín-Nieto J, unpublished). Furthermore, mRNA expression of the six genes above and of *POMGNT2* was detected by our group at relatively low levels by means of RNA-Seq in the human retina [112].

There are currently very few studies in the literature on the *POMGNT2* gene and its protein product, the vast majority dealing with its function and mutational effects [41,42,43,45,64,113]. Regarding its expression, the presence of the *POMGNT2* mRNA has been documented in all human adult tissues examined to date, with its highest levels in the pancreas followed by the brain, testis, kidney, skeletal muscle, prostate and other organs [42]. However, *POMGNT2* expression has not been studied at the protein level, except for its subcellular distribution in the epithelial cell line HEK293, derived from human embryonic kidney [39,43,44], and HeLa cells, derived from a human cervical adenocarcinoma [45].

In the present work, we have evidenced for the first time expression of the POMGNT2 protein in the neural retina of the monkey, cow, rat and mouse, together with the 661W photoreceptor cell line. The presence of POMGNT2 was revealed by immunoblotting in the neural retina of all the species analyzed and in 661W cells, in all cases with an *M*r of ca. 67 kDa, in agreement with its predicted size based on the amino acid sequences of POMGNT2 from the four species studied. Additionally, in the monkey retina a second, higher-size band was observed, with an *M*r of ca. 80 kDa, which exhibited a lower level of expression than the 67 kDa band corresponding to canonical POMGNT2. Likewise, it was determined that POMGNT2 was present in the two subcellular fractions analyzed, i.e., cytoplasmic and nuclear, in the mouse retina and in 661W cells, where the 67 kDa main-protein band and the second, fainter 80 kDa band, were both detected. Furthermore, a third band of ca. 76 kDa was noticeable in the cytoplasmic fraction of the mouse retina—albeit at lower intensity than the 80 and 67 kDa bands—and in both cytoplasmic and nuclear fractions of the 661W cell line. The appearance of the two, 80 and 76 kDa POMGNT2 bands in addition to the canonical 67 kDa isoform, we believe was likely attributable to alternative splicing of the POMGNT2 mRNA and/or post-translational modifications of the POMGNT2 protein. In this context, POMGNT2 has been detected to be *O*-glycosylated at aa 43 in human cultured HEK293 cells and the hepatocellular HepG2 carcinoma cell line [93], to be *N*-glycosylated at two sites, corresponding to aas 276 and 543, in some mouse tissues [92], and to be ubiquitinated at residue 145 in the human Jurkat cell line derived from T lymphocytes [94]. Additionally, the POMGNT2 sequence has been predicted to contain two potential *O*-glycosylation sites, one *N*-glycosylation site and eight phosphorylation sites [92,93,94], all of which could provide a plausible explanation for the origin of the POMGNT2 80 and 76 kDa extra bands detected in our immunoblots of proteins from the mouse retina and/or the 661W cell line.

### 4.2. Immunolocalization of POMGNT2 in the Monkey and Mouse Neural Retinas, and in the 661W Photoreceptor Cell Line

By means of immunohistochemical and immunocytochemical studies coupled with confocal fluorescence microscopy, we have addressed in this work the localization of POMGNT2 protein in the different layers of the neural retina of adult monkeys and mice, as well as its subcellular distribution in the 661W photoreceptor cell line. As a result, we located POMGNT2 in both monkey and mouse retinas in the outer (OS) and inner (IS) segments of photoreceptors, in the OPL, the INL and the GCL. However, in the monkey retina POMGNT2 was additionally present in some nuclei of cells in the INL and in all cell nuclei in the GCL, while in the mouse retina it appeared to be restricted to the nuclei of ganglion cells. Likewise, in this work we have demonstrated for the first time the presence of POMGNT2 in the ER within the IS of photoreceptors, of resident cells in the INL and of ganglion cells in both the monkey and mouse retinas. However, it must be noted that the presence of POMGNT2 in the mouse retina was evident in the Golgi complex, in addition to the ER, of ganglion cells only. These findings corroborated our results obtained by immunoblotting in both mammalian retinas (including both cytoplasmic and nuclear fractions in the mouse).

Additionally, we were able to immunolocate POMGNT2 not only in the cytoplasm, but also in the nucleus of 661W photoreceptors, also in coherence with our results obtained by immunoblotting, in which this protein was detected in the two subcellular fractions. Notably, however, the nuclear presence of POMGNT2 in 661W cells contrasted with its distribution in the monkey and mouse retinas, where POMGNT2 was found absent from the nuclei of (native) photoreceptors. Also, within the cytoplasm of 661W cells POMGNT2 was observed to concentrate in the ER and Golgi complex. These results were consistent with our observation of this protein being present in the ER of photoreceptors and other retinal cells residing in the INL and GCL of both monkey and mouse retinas, as evidenced by our immunolocalization assays. On the other hand, the accumulation of POMGNT2 in the Golgi complex of 661W cells was at variance with its distribution in the retina of both species, where it had not been detected in the Golgi complex of photoreceptors. It is also worth mentioning that the presence of POMGNT2 in the ER and/or the Golgi complex, depending on the species and cell type, was coherent with the presence of a signal peptide in such protein (aas 1–16), as revealed by the InterPro and NCBI Entrez Protein databases, which could be presumed to be responsible for its targeting to both intracellular organelles.

The concentration of POMGNT2 in the ER of the aforementioned retinal cells was consistent with results previously obtained in other studies where POMGNT2 had been localized to the ER of the HEK293 cell line [39,43], of HeLa cells [45] and of the Neuro-2a cell line, derived from mouse neuroblastoma cells [44]. Also, the partial localization of POMGNT2 in the Golgi complex, in addition to the ER, of ganglion cells in the mouse retina and of cultured 661W photoreceptors, was in keeping with a previous report in HEK293 cells [43]. However, it must be emphasized that POMGNT2 is not the first glycosyltransferase involved in the α-DG glycosylation pathway to be found localized simultaneously in both organelles in a particular cell type. In fact, the glycosyltransferases fukutin/FKTN and FKRP are transmembrane proteins that act sequentially, usually in the Golgi complex, to transfer two ribitol-5-phosphate units in tandem onto the core M3 *O*-mannosyl glycan of α-DG using CDP-ribitol as the donor substrate [114,115,116]. In this light, fukutin has been localized to the Golgi of mouse liver tissue cells [114], human HEK293 cells [117,118], COS-7 monkey kidney fibroblasts, NRK cells derived from rat kidney epithelium [119] and C2C12 mouse myoblasts [120]. However, fukutin has also been detected in both the ER and Golgi of human astrocytoma 1321N1 and HeLa cells [121,122], whereas our group reported its localization solely in the ER, but not in the Golgi, of photoreceptors in the mouse neural retina and in 661W cells [85]. The fact that fukutin exhibits a distribution in retinal cells different to that observed in other human cell types suggests that the α-DG glycosylation pathway and/or fukutin function may be in some way distinct in the retina, compared to other tissues. This could also explain why POMGNT2 has been found in the Golgi, additionally to the ER, in several retinal cell types. By contrast, FKRP has been localized to the Golgi complex in a variety of cell types and tissues, including human skeletal muscle [120,123], mouse liver tissue [114], human HEK293 [117,118], monkey COS-7 and rat NRK cells [119], mouse C2C12 cells [120,124,125], Chinese hamster ovary CHO cells [125], human neuroblastoma SHSY-5Y cells, human oligodendrocytic glial hybrid MO3.13 cells and rat H9c2 cardiomyoblasts [126], and -by our group- in the Golgi of mouse photoreceptors and cells residing in both the INL and GCL of the mouse retina, as well as of the 661W cell line [85]. However, mutant FKRP variants have been reported to be abnormally retained in the ER of C2C12 and CHO cells [120,125]. In this context, it must be noted that in the GeneCards database it is predicted, by using the PSORT algorithm, that POMGNT2 could be secreted into the extracellular environment. Therefore, a possible explanation for the normal localization of POMGNT2 in both the Golgi complex and the ER of retinal cells would be that this protein could travel through the endomembrane system derived from these two organelles to be subsequently secreted into the extracellular medium through exocytic vesicles and finally associate with the DGC at this location.

In this work, we also observed the presence of POMGNT2 in the nucleus of particular cell types in the monkey and mouse retinas, as well as in the 661W photoreceptor cell line, prompting further investigation into its potential function(s) within the nuclear compartment. In this light, by means of double immunolabeling experiments we studied the localization of POMGNT2 in a number of nucleoplasmic structures where different nuclear functions take place, namely: (i) euchromatin, (ii) heterochromatin, (iii) nucleolus, (iv) PML nuclear bodies, (v) nuclear Cajal bodies, and (vi) spliceosomes. Our results suggest that POMGNT2 is associated with chromatin, including both euchromatin and heterochromatin, PML and Cajal nuclear bodies, and spliceosomes, being thus absent from nucleoli. All these nucleoplasmic structures in which POMGNT2 accumulated had in common an involvement in transcriptional regulation, particularly active or silenced gene expression (euchromatin or heterochromatin, respectively), especially modulation of transcription (PML bodies), snRNP synthesis and mRNA transcription, processing and splicing (Cajal bodies and spliceosomes). Therefore, these results strongly suggested a function for POMGNT2 in the regulation of gene expression at the mRNA level, both pre- and post-transcriptionally. Consequently, and in an attempt to shed some light into the possible function(s) of POMGNT2 in this regulation, we set out to analyze, using the STRING database of known and predicted protein–protein interactions (https://string-db.org), the possible functional interactions of POMGNT2 with other, non-DG-related nuclear proteins. However, this analysis just revealed that this protein would presumably interact only with DG itself and/or with other glycosyltransferases involved in the α-DG *O*-mannosyl glycosylation pathway (Supplementary Figure S1A).

### 4.3. Immunolocalization of POMGNT1 in the Monkey Neural Retina and 661W Photoreceptor Cell Line

By using immunohistochemistry combined with confocal fluorescence microscopy, we have also addressed in this work the distribution of the POMGNT1 protein in the different layers of the monkey neural retina, as well as its localization at the subcellular level in this tissue and in 661W photoreceptors. In this fashion, we located POMGNT1 in the IS of monkey photoreceptors, as well as in the OPL and around (but not inside) the nuclei of cells located in the INL. Also, we evidenced the presence of POMGNT1 in Golgi complexes located in the monkey IS, OPL and INL, belonging to both photoreceptors and cells residing in the INL. These results illustrated that this protein was expressed in a greater number of layers in the monkey than in the mouse retina since, as reported by our group [63] and others [127], in the mouse POMGNT1 was present solely in the IS. However, these results were consistent with the POMGNT1 expression band detected by immunoblotting in the monkey retina, as well as with its localization in the Golgi complex of photoreceptors detected by immunohistochemistry in the mouse retina, as we previously reported by our group [63].

We also located POMGNT1, by means of immunocytochemistry with a new antibody not previously used in our laboratory, in both the cytoplasm and nucleus of 661W cells, this being consistent with the expression bands of this protein in both subcellular fractions previously obtained by immunoblotting by our research group [63]. However, the detection of POMGNT1 in the nucleus of 661W cells contrasted with our observations in the monkey and mouse retinas in the present work, where this protein was absent from the nuclei of photoreceptors. Also, we have reported that within the cytoplasm of 661W cells POMGNT1 was located in the Golgi complex [63]. These results were consistent with the location of this protein to the Golgi of photoreceptors and cells within the INL of the monkey retina, as revealed by our immunolabeling assays in the present study, as well as with its localization in the Golgi of photoreceptors in the mouse retina and 661W cells, as previously reported by our group [63]. These results were reinforced by previous reports from other groups showing POMGNT1 localization in the Golgi complex of both C2C12 [61] and HeLa cell lines [62], following their transfection with the human *POMGNT1* gene.

The presence of POMGNT1 in the nucleus of the 661W photoreceptor cell line also led us to analyze the possible function of such protein in this subcellular compartment. Specifically, the intranuclear localization of POMGNT1 was assessed in the euchromatin, heterochromatin, nucleolus, PML and Cajal nuclear bodies, and nuclear speckles. As a result, we determined that POMGNT1 was located in the euchromatin, heterochromatin, PML and Cajal nuclear bodies, and spliceosomes, which allowed us to presume, following the same rationale as for POMGNT2 (see above), a possible function for this protein in the regulation of gene expression at the mRNA level, both pre- and post-transcriptionally. Consequently, and in order to test this possibility, we analyzed the putative functional interactions of POMGNT1 with other nuclear proteins using the STRING database of protein–protein interactions. Unfortunately, after carrying out this analysis -and similarly to POMGNT2- it was also obtained that POMGNT1 was functionally related only to DG and other glycosyltransferases involved in the *O*-mannosyl glycosylation pathway of its α subunit, as well as to agrin (Supplementary Figure S1B), an ECM protein known to be present in the OPL of, at least, the mature avian retina [128].

### 4.4. Coimmunolabeling of POMGNT2 and POMGNT1 in the Monkey and Mouse Neural Retinas, and 661W Photoreceptor Cell Line

Once analyzed separately the distribution of POMGNT2 and POMGNT1 proteins in the different layers of the neural retina of adult monkeys and mice, we set out to jointly analyze the expression patterns of the two proteins in both mammalian species. In the monkey retina, POMGNT2 was observed to accumulate in the OS and IS of photoreceptors, in the nuclear layers INL and GCL, and in the two plexiform layers OPL and IPL. Regarding POMGNT1, it was also detected in the IS of photoreceptors, as well as in the OPL, and in the INL and GCL nuclear layers. These two proteins, POMGNT2 and POMGNT1, were thus coexpressed in the IS of photoreceptors, as well as in these three layers, in the monkey retina. However, it was noticeably found that POMGNT2 and POMGNT1 did not colocalize in any of these layers. Regarding the mouse retina, POMGNT2 was visualized in the OS and IS of photoreceptors, in the nuclear layers INL and GCL, and in the two plexiform layers OPL and IPL, while POMGNT1 was visualized solely in the IS of photoreceptors. Therefore, the expression patterns of the two proteins only coincided in the IS of mouse photoreceptors, although no colocalization was either detected between them in this layer. In this light, we determined that POMGNT2 and POMGNT1 were located in different cytoplasmic organelles, the ER and Golgi complex, respectively, in both mammalian retinas examined. Also of note, POMGNT2 had a broader distribution than POMGNT1 across the different layers of the retina in both species. This fact suggested that α-DG could be *O*-mannosyl glycosylated differently depending on the retinal cell type. Alternatively, POMGNT2 could be involved in other intracellular processes, in addition to the α-DG glycosylation pathway, in which POMGNT1 would not participate, an issue that would be much interesting to elucidate.

Following the logical order that protein glycosylation begins in the ER and ends in the Golgi complex, it is widely represented in the literature that, within the myoid portion of the IS of mammalian photoreceptors, the Golgi complex is located above the ER, the latter being the closest organelle to the nuclei of these neurons in the ONL [129,130,131,132,133,134,135,136,137,138,139,140,141,142,143]. However, after having assessed in this work the possible coexpression of POMGNT2 and POMGNT1 in the different layers of the monkey and mouse retinas by means of coimmunolabeling experiments, we noticed that within the IS of photoreceptors the POMGNT2 protein, located in the ER, was above POMGNT1, located in the Golgi, in both mammalian retinas. This led us, given that this double immunostaining had not been performed previously, and in order to verify the relative localization of both organelles within the IS, to jointly immunolabel the ER and the Golgi in the monkey and mouse retinas. As a result, the signals from two separate color strips could be observed within the IS, so that the upper strip corresponded to the ER and the lower strip to the Golgi, in both mammals. Although this was in disagreement with previous literature cited above, in a recent study the 3D organization of mitochondria in primate photoreceptors was documented, and a montage of several micrographs taken by transmission electron microscopy (TEM) of a cone longitudinal section was shown [144]. As illustrated, the ER was widespread throughout the myoid of the IS, while the Golgi was concentrated in the myoid area closest to the cone nucleus [144]. These findings clearly supported the results obtained in our present work, with the ER being positioned above the Golgi complex within the myoid region of the photoreceptors’ IS, in both the monkey and mouse retinas.

Finally, regarding the 661W cell line, POMGNT2 and POMGNT1 were shown to be immunolocated together in both the cytoplasm and the nucleus, with the POMGNT2 expression signal in the cytoplasm being more widespread than that of POMGNT1, and with a high colocalization being found between both proteins in the cytoplasm and nucleus of 661W cells. The colocalization of POMGNT2 and POMGNT1 in the cytoplasm reflected the presence of the two proteins in the Golgi complex, whereas their colocalization in the nucleus was coherently related to their simultaneous association with chromatin (including both euchromatin and heterochromatin), nuclear PML and Cajal bodies and spliceosomes. This leads us to postulate a presumptive indirect role for POMGNT2 and POMGNT1 in the modulation of gene expression at the mRNA level in retinal transformed cells that could be exerted both pre- and post-transcriptionally through the O-GlcNAcylation of nuclear proteins to be identified. This is an interesting possibility that we believe deserves further investigation.

## Figures and Tables

**Table 1:** Primary antibodies used in this work.

Protein	Antibody	Company	Catalog No.	Working Dilution		
				IB	IHC	ICC
POMGNT2	Rabbit, polyclonal	Thermo Fisher Scientific ^a^	PA5-43262	1:500	–	–
POMGNT2	Rabbit, polyclonal	Signalway Antibody ^b^	38956	–	1:100	1:100
POMGNT1	Mouse, clone 6C12	Sigma-Aldrich ^c^	WH0055624M7	–	1:50	1:50
POMGNT1	Rabbit, polyclonal	Thermo Fisher Scientific	PA5-76448	–	1:100	1:100
β-Tubulin III	Rabbit, polyclonal	Sigma-Aldrich	T3952	1:500	–	–
Lamina A/C	Mouse, clone 4C11	Sigma-Aldrich	SAB200236	1:1000	–	–
KDEL	Mouse, clone 10C3	Abcam ^d^	ab12223	–	1:100	1:100
GM130	Mouse, clone 35	BD Transduction Laboratories ^e^	610822	–	1:100	1:100
GM130	Rabbit, polyclonal	Thermo Fisher Scientific	PA5-85643	–	1:100	–

List of antibodies used in this study, for which detailed information is provided, including the antigen, species of origin, clone code for monoclonal antibodies, commercial company, catalogue reference number, and dilution at which each antibody was used for immunoblotting (IB), immunohistochemistry (IHC) or immunocytochemistry (ICC) experiments. ^a^ Thermo Fisher Scientific (Waltham, MA, USA), ^b^ Signalway Antibody (Greenbelt, MD, USA), ^c^ Sigma-Aldrich (Saint Louis, MO, USA), ^d^ Abcam (Cambridge, UK), ^e^ BD Transduction Laboratories (Franklin Lakes, NJ, USA).

## Data Availability

All relevant data are contained within this article.

## Supplementary Materials

The following supporting information can be downloaded at: https://www.mdpi.com/article/10.3390/biomedicines13112759/s1, Figure S1: Networks of (known and predicted) protein-protein interactions generated by the STRING (v12.0) webtool for POMGNT2 (A) and POMGNT1 (B) proteins, both boxed in orange.

## Abbreviations

The following abbreviations are used in this manuscript:

DGPs	Dystroglycanopathies
DG	Dystroglycan
ER	Endoplasmic reticulum
CNS	Central nervous system
ECM	Extracellular matrix
DGC	Dystrophin-glycoprotein complex
OPL	Outer plexiform layer
POMGNT2	Protein *O*-linked-mannose β-1,4-*N*-acetylglucosaminyltransferase 2
POMGNT1	Protein *O*-linked-mannose β-1,2-*N*-acetylglucosaminyltransferase 1
KO	Knockout
PB	Sodium phosphate buffer
IB	Immunoblotting
IHC	Immunohistochemistry
ICC	Immunocytochemistry
PBX	Triton X-100
DAPI	4’,6-Diamidino-2-phenylindole
MCC	Manders colocalization coefficient
SD	Standard deviation
DPBS	Dulbecco’s PBS
IS	Inner segment
OS	Outer segment
INL	Inner nuclear layer
GCL	Ganglion cell layer
IPL	Inner plexiform layer
H3K4me3	Histone H3 trimethylated at amino acid Lys-4
PML	Promyelocytic leukemia nuclear bodies
snRNPs	Small nuclear ribonucleoproteins
FKTN	Fukutin
FKRP	Fukutin-related protein
aa	Amino acid

## References

[1] Muntoni F., Brockington M., Torelli S., Brown S.C. (2004). Defective Glycosylation in Congenital Muscular Dystrophies. Curr. Opin. Neurol..

[2] Martin P.T. (2007). Congenital Muscular Dystrophies Involving the *O*-Mannose Pathway. Curr. Mol. Med..

[3] Hewitt J.E. (2009). Abnormal Glycosylation of Dystroglycan in Human Genetic Disease. Biochim. Biophys. Acta.

[4] Muntoni F., Torelli S., Wells D.J., Brown S.C. (2011). Muscular Dystrophies Due to Glycosylation Defects: Diagnosis and Therapeutic Strategies. Curr. Opin. Neurol..

[5] Dobson C.M., Hempel S.J., Stalnaker S.H., Stuart R., Wells L. (2013). *O*-Mannosylation and Human Disease. Cell. Mol. Life Sci..

[6] Jahncke J.N., Wright K.M. (2023). The Many Roles of Dystroglycan in Nervous System Development and Function: Dystroglycan and Neural Circuit Development. Dev. Dyn..

[7] Muntoni F., Voit T. (2004). The Congenital Muscular Dystrophies in 2004: A Century of Exciting Progress. Neuromuscul. Disord..

[8] Schessl J., Zou Y., Bönnemann C.G. (2006). Congenital Muscular Dystrophies and the Extracellular Matrix. Semin. Pediatr. Neurol..

[9] Reed U. (2009). Congenital Muscular Dystrophy. Part I: A Review of Phenotypical and Diagnostic Aspects. Arq. Neuropsiquiatr..

[10] Godfrey C., Foley A.R., Clement E., Muntoni F. (2011). Dystroglycanopathies: Coming Into Focus. Curr. Opin. Genet. Dev..

[11] Mercuri E., Muntoni F. (2012). The Ever-Expanding Spectrum of Congenital Muscular Dystrophies. Ann. Neurol..

[12] Smalheiser N.R., Schwartz N.B. (1987). Cranin: A Laminin Binding Protein of Cell Membranes. Proc. Natl. Acad. Sci. USA.

[13] Ibraghimov-Beskrovnaya O., Ervasti J.M., Leveille C.J., Slaughter C.A., Sernett S.W., Campbell K.P. (1992). Primary Structure of Dystrophin-Associated Glycoproteins Linking Dystrophin to the Extracellular Matrix. Nature.

[14] Ibraghimov-Beskrovnaya O., Milatovich A., Ozcelik T., Yang B., Koepnick K., Francke U., Campbell K.P. (1993). Human Dystroglycan: Skeletal Muscle cDNA, Genomic Structure, Origin of Tissue Specific Isoforms and Chromosomal Localization. Hum. Mol. Genet..

[15] Durbeej M. (2010). Laminins. Cell Tissue Res..

[16] Durbeej M., Larsson E., Ibraghimov-Beskrovnaya O., Roberds S.L., Campbell K.P., Ekblom P. (1995). Non-Muscle α-Dystroglycan is Involved in Epithelial Development. J. Cell Biol..

[17] Durbeej M., Henry M.D., Ferletta M., Campbell K.P., Ekblom P. (1998). Distribution of Dystroglycan in Normal Adult Mouse Tissues. J. Histochem. Cytochem..

[18] Durbeej M., Campbell K.P. (1999). Biochemical Characterization of the Epithelial Dystroglycan Complex. J. Biol. Chem..

[19] Esser A.K., Cohen M.B., Henry M.D. (2010). Dystroglycan Is Not Required for Maintenance of the Luminal Epithelial Basement Membrane or Cell Polarity in the Mouse Prostate. Prostate.

[20] Holt K.H., Crosbie R.H., Venzke D.P., Campbell K.P. (2000). Biosynthesis of Dystroglycan: Processing of a Precursor Propeptide. FEBS Lett..

[21] Akhavan A., Crivelli S.N., Singh M., Lingappa V.R., Muschler J.L. (2008). SEA Domain Proteolysis Determines the Functional Composition of Dystroglycan. FASEB J..

[22] Oppizzi M.L., Akhavan A., Singh M., Fata J.E., Muschler J.L. (2008). Nuclear Translocation of β-Dystroglycan Reveals a Distinctive Trafficking Pattern of Autoproteolyzed Mucins. Traffic.

[23] Ervasti J.M., Campbell K.P. (1991). Membrane Organization of the Dystrophin-Glycoprotein Complex. Cell.

[24] Brancaccio A., Schulthess T., Gesemann M., Engel J. (1995). Electron Microscopic Evidence for a Mucin-Like Region in Chick Muscle α-Dystroglycan. FEBS Lett..

[25] Brancaccio A., Schulthess T., Gesemann M., Engel J. (1997). The N-Terminal Region of α-Dystroglycan Is an Autonomous Globular Domain. Eur. J. Biochem..

[26] Winder S.J. (2001). The Complexities of Dystroglycan. Trends Biochem. Sci..

[27] Barresi R., Campbell K.P. (2006). Dystroglycan: From Biosynthesis to Pathogenesis of Human Disease. J. Cell Sci..

[28] Samwald M. (2007). Review: Dystroglycan in the Nervous System. Nat. Preced..

[29] Sato S., Omori Y., Katoh K., Kondo M., Kanagawa M., Miyata K., Funabiki K., Koyasu T., Kajimura N., Miyoshi T. (2008). Pikachurin, a Dystroglycan Ligand, Is Essential for Photoreceptor Ribbon Synapse Formation. Nat. Neurosci..

[30] Nickolls A.R., Bönnemann C.G. (2018). The Roles of Dystroglycan in the Nervous System: Insights From Animal Models of Muscular Dystrophy. Dis. Models Mech..

[31] Huang X., Poy F., Zhang R., Joachimiak A., Sudol M., Eck M.J. (2000). Structure of a WW Domain Containing Fragment of Dystrophin in Complex With β-Dystroglycan. Nat. Struct. Biol..

[32] Bozzi M., Morlacchi S., Bigotti M.G., Sciandra F., Brancaccio A. (2009). Functional Diversity of Dystroglycan. Matrix Biol..

[33] Endo T. (2015). Glycobiology of α-Dystroglycan and Muscular Dystrophy. J. Biochem..

[34] Ragni E., Lommel M., Moro M., Crosti M., Lavazza C., Parazzi V., Saredi S., Strahl S., Lazzari L. (2016). Protein *O*-Mannosylation Is Crucial for Human Mesenchymal Stem Cells Fate. Cell. Mol. Life Sci..

[35] Endo T. (2019). Mammalian *O*-Mannosyl Glycans: Biochemistry and Glycopathology. Proc. Jpn. Acad. Ser. B Phys. Biol. Sci..

[36] Hino N., Kobayashi M., Shibata N., Yamamoto T., Saito K., Osawa M. (2001). Clinicopathological Study on Eyes From Cases of Fukuyama Type Congenital Muscular Dystrophy. Brain Dev..

[37] Lee Y., Kameya S., Cox G.A., Hsu J., Hicks W., Maddatu T.P., Smith R.S., Naggert J.K., Peachey N.S., Nishina P.M. (2005). Ocular Abnormalities in *Largemyd* and *Largevls* Mice, Spontaneous Models for Muscle, Eye, and Brain Diseases. Mol. Cell. Neurosci..

[38] Hu H., Candiello J., Zhang P., Ball S.L., Cameron D.A., Halfter W. (2010). Retinal Ectopias and Mechanically Weakened Basement Membrane in a Mouse Model of Muscle-Eye-Brain (MEB) Disease Congenital Muscular Dystrophy. Mol. Vis..

[39] Yoshida-Moriguchi T., Willer T., Anderson M.E., Venzke D., Whyte T., Muntoni F., Lee H., Nelson S.F., Yu L., Campbell K.P. (2013). SGK196 Is a Glycosylation-Specific *O*-Mannose Kinase Required for Dystroglycan Function. Science.

[40] Yoshida-Moriguchi T., Campbell K.P. (2015). Matriglycan: A Novel Polysaccharide That Links Dystroglycan to the Basement Membrane. Glycobiology.

[41] Halmo S.M., Singh D., Patel S., Wang S., Edlin M., Boons G.J., Moremen K.W., Live D., Wells L. (2017). Protein *O*-Linked Mannose β-1,4-*N*-Acetylglucosaminyltransferase 2 (POMGNT2) Is a Gatekeeper Enzyme for Functional Glycosylation of α-Dystroglycan. J. Biol. Chem..

[42] Manzini M.C., Tambunan D.E., Hill R.S., Yu T.W., Maynard T.M., Heinzen E.L., Shianna K.V., Stevens C.R., Partlow J.N., Barry B.J. (2012). Exome Sequencing and Functional Validation in Zebrafish Identify *GTDC2* Mutations as a Cause of Walker-Warburg Syndrome. Am. J. Hum. Genet..

[43] Ogawa M., Nakamura N., Nakayama Y., Kurosaka A., Manya H., Kanagawa M., Endo T., Furukawa K., Okajima T. (2013). GTDC2 Modifies *O*-mannosylated α-Dystroglycan in the Endoplasmic Reticulum to Generate *N*-Acetyl Glucosamine Epitopes Reactive with CTD110.6 Antibody. Biochem. Biophys. Res. Commun..

[44] Yagi H., Nakagawa N., Saito T., Kiyonari H., Abe T., Toda T., Wu S., Khoo K., Oka S., Kato K. (2013). AGO61-Dependent GlcNAc Modification Primes the Formation of Functional Glycans on α-Dystroglycan. Sci. Rep..

[45] Endo Y., Dong M., Noguchi S., Ogawa M., Hayashi Y.K., Kuru S., Sugiyama K., Nagai S., Ozasa S., Nonaka I. (2015). Milder Forms of Muscular Dystrophy Associated with *POMGNT2* Mutations. Neurol. Genet..

[46] Stalnaker S.H., Aoki K., Lim J.M., Porterfield M., Liu M., Satz J.S., Buskirk S., Xiong Y., Zhang P., Campbell K.P. (2011). Glycomic Analyses of Mouse Models of Congenital Muscular Dystrophy. J. Biol. Chem..

[47] Praissman J.L., Wells L. (2014). Mammalian *O*-Mannosylation Pathway: Glycan Structures, Enzymes, and Protein Substrates. Biochemistry.

[48] Yoshida-Moriguchi T., Yu L., Stalnaker S., Sarah Davis S.H., Kunz S., Madson M., Oldstone M.B.A., Schachter H., Wells L., Campbell K.P. (2010). *O*-Mannosyl Phosphorylation of Alpha-Dystroglycan Is Required for Laminin Binding. Science.

[49] Beedle A.M., Turner A.J., Saito Y., Lueck J.D., Foltz S.J., Fortunato M.J., Nienaber P.M., Campbell K.P. (2012). Mouse *fukutin* Deletion Impairs Dystroglycan Processing and Recapitulates Muscular Dystrophy. J. Clin. Investig..

[50] Inamori K.I., Yoshida-Moriguchi T., Hara Y., Anderson M.E., Yu L., Campbell K.P. (2012). Dystroglycan Function Requires Xylosyl-and Glucuronyltransferase Activities of LARGE. Science.

[51] Kuga A., Kanagawa M., Sudo A., Chan Y.M., Tajiri M., Manya H., Kikkawa Y., Nomizu M., Kobayashi K., Endo T. (2012). Absence of Post-Phosphoryl Modification in Dystroglycanopathy Mouse Models and Wild-Type Tissues Expressing Non-Laminin Binding Form of α-Dystroglycan. J. Biol. Chem..

[52] Bao X., Kobayashi M., Hatakeyama S., Angata K., Gullberg D., Nakayama J., Fukuda M.N., Fukuda M. (2009). Tumor Suppressor Function of Laminin-Binding α-Dystroglycan Requires a Distinct β3-*N*-Acetylglucosaminyltransferase. Proc. Natl. Acad. Sci. USA.

[53] Beltrán-Valero de Bernabé D., Inamori K.I., Yoshida-Moriguchi T., Weydert C.J., Harper H.A., Willer T., Henry M.D., Campbell K.P. (2009). Loss of α-Dystroglycan Laminin Binding in Epithelium-Derived Cancers Is Caused by Silencing of *LARGE*. J. Biol. Chem..

[54] Tsuboi S., Hatakeyama S., Ohyama C., Fukuda M. (2012). Two Opposing Roles of *O*-Glycans in Tumor Metastasis. Trends Mol. Med..

[55] Esser A.K., Miller M.R., Huang Q., Meier M.M., Beltrán-Valero de Bernabé D., Stipp C.S., Campbell K.P., Lynch C.F., Smith B.J., Cohen M.B. (2013). Loss of LARGE2 Disrupts Functional Glycosylation of α-Dystroglycan in Prostate Cancer. J. Biol. Chem..

[56] Quereda C., Pastor À., Martín-Nieto J. (2022). Involvement of Abnormal Dystroglycan Expression and Matriglycan Levels in Cancer Pathogenesis. Cancer Cell Int..

[57] Yoshida A., Kobayashi K., Manya H., Taniguchi K., Kano H., Mizuno M., Inazu T., Mitsuhashi H., Takahashi S., Takeuchi M. (2001). Muscular Dystrophy and Neuronal Migration Disorder Caused by Mutations in a Glycosyltransferase, POMGnT1. Dev. Cell.

[58] Satz J.S., Ostendorf A.P., Hou S., Turner A., Kusano H., Lee J.C., Turk R., Nguyen H., Ross-Barta S.E., Westra S. (2010). Distinct Functions of Glial and Neuronal Dystroglycan in the Developing and Adult Mouse Brain. J. Neurosci..

[59] Wright K.M., Lyon K.A., Leung H., Leahy D.J., Ma L., Ginty D.D. (2012). Dystroglycan Organizes Axon Guidance Cue Localization and Axonal Pathfinding. Neuron.

[60] Zhang W., Betel D., Schachter H. (2002). Cloning and Expression of a Novel UDP-GlcNAc:α-D-Mannoside β1,2-*N*-Acetylglucosaminyltransferase Homologous to UDP-GlcNAc:α-3-D-mannoside β1,2-*N*-acetylglucosaminyltransferase I. Biochem. J..

[61] Xiong H., Kobayashi K., Tachikawa M., Manya H., Takeda S., Chiyonobu T., Fujikake N., Wang F., Nishimoto A., Morris G.E. (2006). Molecular Interaction Between Fukutin and POMGnT1 in the Glycosylation Pathway of α-Dystroglycan. Biochem. Biophys. Res. Commun..

[62] Pereira N.A., Pu H.X., Goh H., Song Z. (2014). Golgi Phosphoprotein 3 Mediates the Golgi Localization and Function of Protein *O*-Linked Mannose β-1,2-*N*-Acetlyglucosaminyltransferase 1. J. Biol. Chem..

[63] Uribe M.L., Haro C., Ventero M.P., Campello L., Cruces J., Martín-Nieto J. (2016). Expression Pattern in Retinal Photoreceptors of POMGnT1, a Protein Involved in Muscle-Eye-Brain Disease. Mol. Vis..

[64] Nakagawa N., Yagi H., Kato K., Takematsu H., Oka S. (2015). Ectopic Clustering of Cajal-Retzius and Subplate Cells is an Initial Pathological Feature in *Pomgnt2*-Knockout Mice, a Model of Dystroglycanopathy. Sci. Rep..

[65] Liu J., Ball S.L., Yang Y., Mei P., Zhang L., Shi H., Kaminski H.J., Lemmon V.P., Hu H. (2006). A Genetic Model for Muscle–Eye–Brain Disease in Mice Lacking Protein *O*-Mannose 1,2-*N*-Acetylglucosaminyltransferase (POMGnT1). Mech. Dev..

[66] Hu H., Yang Y., Eade A., Xiong Y., Qi Y. (2007). Breaches of the Pial Basement Membrane and Disappearance of the Glia Limitans During Development Underlie the Cortical Lamination Defect in the Mouse Model of Muscle-Eye-Brain Disease. J. Comp. Neurol..

[67] Yang Y., Zhang P., Xiong Y., Li X., Qi Y., Hu H. (2007). Ectopia of Meningeal Fibroblasts and Reactive Gliosis in the Cerebral Cortex of the Mouse Model of Muscle-Eye-Brain Disease. J. Comp. Neurol..

[68] Miyagoe-Suzuki Y., Masubuchi N., Miyamoto K., Wada M.R., Yuasa S., Saito F., Matsumura K., Kanesaki H., Kudo A., Manya H. (2009). Reduced Proliferative Activity of Primary *POMGnT1*-Null Myoblasts In Vitro. Mech. Dev..

[69] Kanagawa M., Omori Y., Sato S., Kobayashi K., Miyagoe-Suzuki Y., Takeda S., Endo T., Furukawa T., Toda T. (2010). Post-Translational Maturation of Dystroglycan Is Necessary for Pikachurin Binding and Ribbon Synaptic Localization. J. Biol. Chem..

[70] Liu J., Yang Y., Li X., Zhang P., Qi Y., Hu H. (2010). Cellular and Molecular Characterization of Abnormal Brain Development in Protein *O*-Mannose *N*-Acetylglucosaminyltransferase 1 Knockout Mice. Methods Enzymol..

[71] Hu H., Li J., Zhang Z., Yu M. (2011). Pikachurin Interaction with Dystroglycan Is Diminished by Defective *O*-Mannosyl Glycosylation in Congenital Muscular Dystrophy Models and Rescued by LARGE Overexpression. Neurosci. Lett..

[72] Li J., Yu M., Feng G., Hu H., Li X. (2011). Breaches of the Pial Basement Membrane Are Associated with Defective Dentate Gyrus Development in Mouse Models of Congenital Muscular Dystrophies. Neurosci. Lett..

[73] Takahashi H., Kanesaki H., Igarashi T., Kameya S., Yamaki K., Mizota A., Kudo A., Miyagoe-Suzuki Y., Takeda S., Takahashi H. (2011). Reactive Gliosis of Astrocytes and Müller Glial Cells in Retina of POMGnT1-Deficient Mice. Mol. Cell. Neurosci..

[74] Yu M., He Y., Wang K., Zhang P., Zhang S., Hu H. (2013). Adeno-Associated Viral-Mediated *LARGE* Gene Therapy Rescues the Muscular Dystrophic Phenotype in Mouse Models of Dystroglycanopathy. Hum. Gene Ther..

[75] Zhang P., Yang Y., Candiello J., Thorn T.L., Gray N., Halfter W.M., Hu H. (2013). Biochemical and Biophysical Changes Underlie the Mechanisms of Basement Membrane Disruptions in a Mouse Model of Dystroglycanopathy. Matrix Biol..

[76] Booler H.S., Williams J.L., Hopkinson M., Brown S.C. (2016). Degree of Cajal-Retzius Cell Mislocalization Correlates with the Severity of Structural Brain Defects in Mouse Models of Dystroglycanopathy. Brain Pathol..

[77] Morioka S., Sakaguchi H., Mohri H., Taniguchi-Ikeda M., Kanagawa M., Suzuki T., Miyagoe-Suzuki Y., Toda T., Saito N., Ueyama T. (2020). Congenital Hearing Impairment Associated with Peripheral Cochlear Nerve Dysmyelination in Glycosylation-Deficient Muscular Dystrophy. PLoS Genet..

[78] Campello L., Esteve-Rudd J., Bru-Martínez R., Herrero M.T., Fernández-Villalba E., Cuenca N., Martín-Nieto J. (2013). Alterations in Energy Metabolism, Neuroprotection and Visual Signal Transduction in the Retina of Parkinsonian, MPTP-Treated Monkeys. PLoS ONE.

[79] Esteve-Rudd J., Fernández-Sánchez L., Lax P., De Juan E., Martín-Nieto J., Cuenca N. (2011). Rotenone Induces Degeneration of Photoreceptors and Impairs the Dopaminergic System in the Rat Retina. Neurobiol. Dis..

[80] Brunet A.A., James R.E., Swanson P., Carvalho L.S. (2025). A Review of the 661W Cell Line as a Tool to Facilitate Treatment Development for Retinal Diseases. Cell Biosci..

[81] Al-Ubaidi M.R., Font R.L., Quiambao A.B., Keener M.J., Liou G.I., Overbeek P.A., Baehr W. (1992). Bilateral Retinal and Brain Tumors in Transgenic Mice Expressing Simian Virus 40 Large T Antigen Under Control of the Human Interphotoreceptor Retinoid-Binding Protein promoter. J. Cell Biol..

[82] Tan E., Ding X.Q., Saadi A., Agarwal N., Naash M.I., Al-Ubaidi M.R. (2004). Expression of Cone-Photoreceptor-Specific Antigens in a Cell Line Derived From Retinal Tumors in Transgenic Mice. Investig. Ophthalmol. Vis. Sci..

[83] Kanan Y., Moiseyev G., Agarwal N., Ma J.X., Al-Ubaidi M.R. (2007). Light Induces Programmed Cell Death by Activating Multiple Independent Proteases in a Cone Photoreceptor Cell Line. Investig. Ophthalmol. Vis. Sci..

[84] Al-Ubaidi M.R., Matsumoto H., Kurono S., Singh A. (2008). Proteomics Profiling of the Cone Photoreceptor Cell Line, 661W. Adv. Exp. Med. Biol..

[85] Haro C., Uribe M.L., Quereda C., Cruces J., Martín-Nieto J. (2018). Expression in Retinal Neurons of Fukutin and FKRP, the Protein Products of Two Dystroglycanopathy-Causative Genes. Mol. Vis..

[86] Martínez-Navarrete G.C., Martín-Nieto J., Esteve-Rudd J., Angulo A., Cuenca N. (2007). α-Synuclein Gene Expression Profile in the Retina of Vertebrates. Mol. Vis..

[87] Combs A.C., Ervasti J.M. (2005). Enhanced Laminin Binding by α-Dystroglycan After Enzymatic Deglycosylation. Biochem. J..

[88] Esteve-Rudd J., Campello L., Herrero M.T., Cuenca N., Martín-Nieto J. (2010). Expression in the Mammalian Retina of Parkin and UCH-L1, Two Components of the Ubiquitin-Proteasome System. Brain Res..

[89] Manders E.M.M., Stap J., Brakenhoff G.J., van Driel R., Aten J.A. (1992). Dynamics of Three-Dimensional Replication Patterns During the S-Phase, Analysed by Double Labelling of DNA and Confocal Microscopy. J. Cell Sci..

[90] Bolte S., Cordelières F.P. (2006). A Guided Tour Into Subcellular Colocalization Analysis in Light Microscopy. J. Microsc..

[91] Luo T., Mitra S., McBride J.W. (2018). *Ehrlichia chaffeensis* TRP75 Interacts with Host Cell Targets Involved in Homeostasis, Cytoskeleton Organization, and Apoptosis Regulation to Promote Infection. mSphere.

[92] Zielinska D.F., Gnad F., Wiśniewski J.R., Mann M. (2010). Precision Mapping of an In Vivo *N*-Glycoproteome Reveals Rigid Topological and Sequence Constraints. Cell.

[93] Steentoft C., Vakhrushev S.Y., Joshi H.J., Kong Y., Vester-Christensen M.B., Schjoldager K.T.B.G., Lavrsen K., Dabelsteen S., Pedersen N.B., Marcos-Silva L. (2013). Precision Mapping of the Human *O*-GalNAc Glycoproteome Through SimpleCell Technology. EMBO J..

[94] Udeshi N.D., Svinkina T., Mertins P., Kuhn E., Mani D.R., Qiao J.W., Carr S.A. (2013). Refined Preparation and Use of Anti-Diglycine Remnant (K-ε-GG) Antibody Enables Routine Quantification of 10,000s of Ubiquitination Sites in Single Proteomics Experiments. Mol. Cell. Proteom..

[95] Napier R.M., Fowke L.C., Hawes C., Lewis M., Pelham H.R.B. (1992). Immunological Evidence that Plants Use Both HDEL and KDEL for Targeting Proteins to the Endoplasmic Reticulum. J. Cell Sci..

[96] Nakamura N., Rabouille C., Watson R., Nilsson T., Hui N., Slusarewicz P., Kreis T.E., Warren G. (1995). Characterization of a *cis*-Golgi Matrix Protein, GM130. J. Cell Biol..

[97] Santos-Rosa H., Schneider R., Bannister A., Sherriff J., Bernstein B., Emre N., Schreiber S., Mellor J., Kouzarides T. (2002). Active Genes are Tri-Methylated at K4 of Histone H3. Nature.

[98] Cowell I.G., Aucott R., Mahadevaiah S.K., Burgoyne P.S., Huskisson N., Bongiorni S., Prantera G., Fanti L., Pimpinelli S., Wu R. (2002). Heterochromatin, HP1 and Methylation at Lysine 9 of Histone H3 in Animals. Chromosoma.

[99] Boisvert F.M., Van Koningsbruggen S., Navascués J., Lamond A.I. (2007). The Multifunctional Nucleolus. Nat. Rev. Mol. Cell Biol..

[100] Ochs R.L., Lischwe M.A., Spohn W.H., Busch H. (1985). Fibrillarin: A New Protein of the Nucleolus Identified by Autoimmune Sera. Biol. Cell.

[101] Tessarz P., Santos-Rosa H., Robson S.C., Sylvestersen K.B., Nelson C.J., Nielsen M.L., Kouzarides T. (2014). Glutamine Methylation in Histone H2A Is an RNA-Polymerase-I-Dedicated Modification. Nature.

[102] Iyer-Bierhoff A., Krogh N., Tessarz P., Ruppert T., Nielsen H., Grummt I. (2018). SIRT7-Dependent Deacetylation of Fibrillarin Controls Histone H2A Methylation and rRNA Synthesis During the Cell Cycle. Cell Rep..

[103] Sahin U., Lallemand-Breitenbach V., De Thé H. (2014). PML Nuclear Bodies: Regulation, Function and Therapeutic Perspectives. J. Pathol..

[104] Borden K.L.B. (2002). Pondering the Promyelocytic Leukemia Protein (PML) Puzzle: Possible Functions for PML Nuclear Bodies. Mol. Cell. Biol..

[105] Gall J.G. (2003). The Centennial of the Cajal Body. Nat. Rev. Mol. Cell Biol..

[106] Carmo-Fonseca M., Ferreira J., Lamond A.I. (1993). Assembly of snRNP-Containing Coiled Bodies Is Regulated in Interphase and Mitosis–Evidence that the Coiled Body Is a Kinetic Nuclear Structure. J. Cell Biol..

[107] Azubel M., Wolf S.G., Sperling J., Sperling R. (2004). Three-Dimensional Structure of the Native Spliceosome by Cryoelectron Microscopy. Mol. Cell.

[108] Will C.L., Lührmann R. (2011). Spliceosome Structure and Function. Cold Spring Harb. Perspect. Biol..

[109] Zhang X., Yan C., Hang J., Finci L.I., Lei J., Shi Y. (2017). An Atomic Structure of the Human Spliceosome. Cell.

[110] Prados B., Peña A., Cotarelo R.P., Valero M.C., Cruces J. (2007). Expression of the Murine *Pomt1* Gene in Both the Developing Brain and Adult Muscle Tissues and its Relationship with Clinical Aspects of Walker-Warburg Syndrome. Am. J. Pathol..

[111] Rubio-Fernández M., Uribe M.L., Vicente-Tejedor J., Germain F., Susín-Lara C., Quereda C., Montoliu L., de la Villa P., Martín-Nieto J., Cruces J. (2018). Impairment of Photoreceptor Ribbon Synapses in a Novel *Pomt1* Conditional Knockout Mouse Model of Dystroglycanopathy. Sci. Rep..

[112] Campello L., Martín-Nieto J. (2013). RNA-Seq Expression Profile of Genes Related to Neurodegenerative Disorders Affecting the Human Retina. EMBnet J..

[113] Yang J.Y., Halmo S.M., Praissman J., Chapla D., Singh D., Wells L., Moremen K.W., Lanzilotta W.N. (2021). Crystal Structures of β-1,4-*N*-Acetylglucosaminyl-Transferase 2: Structural Basis for Inherited Muscular Dystrophies. Acta Crystallogr. D Struct. Biol..

[114] Kanagawa M., Kobayashi K., Tajiri M., Manya H., Kuga A., Yamaguchi Y., Akasaka-Manya K., Furukawa J., Mizuno M., Kawakami H. (2016). Identification of a Post-Translational Modification with Ribitol-Phosphate and Its Defect in Muscular Dystrophy. Cell Rep..

[115] Kanagawa M., Toda T. (2017). Muscular Dystrophy with Ribitol-Phosphate Deficiency: A Novel Post-Translational Mechanism in Dystroglycanopathy. J. Neuromuscul. Dis..

[116] Kanagawa M., Toda T. (2018). Ribitol-Phosphate—A Newly Identified Posttranslational Glycosylation Unit in Mammals: Structure, Modification Enzymes and Relationship to Human Diseases. J. Biochem..

[117] Willer T., Inamori K., Venzke D., Harvey C., Morgensen G., Hara Y., Beltrán Valero de Bernabé D., Yu L., Wright K., Campbell K. (2014). The Glucuronyltransferase B4GAT1 Is Required for Initiation of LARGE-Mediated α-Dystroglycan Functional Glycosylation. ELife.

[118] Nishihara R., Kobayashi K., Imae R., Tsumoto H., Manya H., Mizuno M., Kanagawa M., Endo T., Toda T. (2018). Cell Endogenous Activities of Fukutin and FKRP Coexist with the Ribitol Xylosyltransferase, TMEM5. Biochem. Biophys. Res. Commun..

[119] Esapa C.T., Benson M.A., Schröder J.E., Martin-Rendon E., Brockington M., Brown S.C., Muntoni F., Kröger S., Blake D.J. (2002). Functional Requirements for Fukutin-Related Protein in the Golgi Apparatus. Hum. Mol. Genet..

[120] Matsumoto H., Noguchi S., Sugie K., Ogawa M., Murayama K., Hayashi Y.K., Nishino I. (2004). Subcellular Localization of Fukutin and Fukutin-Related Protein in Muscle Cells. J. Biochem..

[121] Yamamoto T., Kato Y., Shibata N., Sawada T., Osawa M., Kobayashi M. (2008). A Role of Fukutin, a Gene Responsible for Fukuyama Type Congenital Muscular Dystrophy, in Cancer Cells: A Possible Role to Suppress Cell Proliferation. Int. J. Exp. Pathol..

[122] Yamamoto T., Shibata N., Saito Y., Osawa M., Kobayashi M. (2010). Functions of Fukutin, a Gene Responsible for Fukuyama Type Congenital Muscular Dystrophy, in Neuromuscular System and Other Somatic Organs. Cent. Nerv. Syst. Agents Med. Chem..

[123] Alhamidi M., Kjeldsen Buvang E., Fagerheim T., Brox V., Lindal S., Van Ghelue M., Nilssen Ø. (2011). Fukutin-Related Protein Resides in the Golgi Cisternae of Skeletal Muscle Fibres and Forms Disulfide-Linked Homodimers Via an N-Terminal Interaction. PLoS ONE.

[124] Dolatshad N.F., Brockington M., Torelli S., Skordis L., Wever U., Wells D.J., Muntoni F., Brown S.C. (2005). Mutated Fukutin-Related Protein (FKRP) Localises as Wild Type in Differentiated Muscle Cells. Exp. Cell Res..

[125] Keramaris-Vrantsis E., Lu P.J., Doran T., Zillmer A., Ashar J., Esapa C.T., Benson M.A., Blake D.J., Rosenfeld J., Lu Q.L. (2007). Fukutin-Related Protein Localizes to the Golgi Apparatus and Mutations Lead to Mislocalization in Muscle In Vivo. Muscle Nerve.

[126] Torelli S., Brown S.C., Brockington M., Dolatshad N.F., Jimenez C., Skordis L., Feng L.H., Merlini L., Jones D.H., Romero N. (2005). Sub-Cellular Localisation of Fukutin Related Protein in Different Cell Lines and in the Muscle of Patients with MDC1C and LGMD2I. Neuromuscul. Disord..

[127] Xu M., Yamada T., Sun Z., Eblimit A., Lopez I., Wang F., Manya H., Xu S., Zhao L., Li Y. (2016). Mutations in *POMGNT1* Cause Non-Syndromic Retinitis Pigmentosa. Hum. Mol. Genet..

[128] Koulen P., Honig L.S., Fletcher E.L., Kröger S. (1999). Expression, Distribution and Ultrastructural Localization of the Synapse-Organizing Molecule Agrin in the Mature Avian Retina. Eur. J. Neurosci..

[129] Krizaj D., Copenhagen D.R. (2002). Calcium Regulation in Photoreceptors. Front. Biosci..

[130] Liu Q., Tan G., Levenkova N., Li T., Pugh E.N., Rux J.J., Speichers D.W., Pierce E.A. (2007). The Proteome of the Mouse Photoreceptor Sensory Cilium Complex. Mol. Cell. Proteom..

[131] Murray A.R., Fliesler S.J., Al-Ubaidi M.R. (2009). Rhodopsin: The Functional Significance of Asn-Linked Glycosylation and Other Post-Translational Modifications. Ophthalmic Genet..

[132] Perkins B.D., Fadool J.M. (2010). Photoreceptor Structure and Development: Analyses Using *GFP* Transgenes. Methods Cell Biol..

[133] Kroeger H., Chiang W.C., Lin J.H. (2012). Endoplasmic Reticulum-Associated Degradation (ERAD) of Misfolded Glycoproteins and Mutant *P23H* Rhodopsin in Photoreceptor Cells. Adv. Exp. Med. Biol..

[134] Baker S.A., Kerov V. (2013). Photoreceptor Inner and Outer Segments. Curr. Top. Membr..

[135] Baehr W. (2014). Membrane Protein Transport in Photoreceptors: The Function of PDEδ. Investig. Ophthalmol. Vis. Sci..

[136] Cuenca N., Fernández-Sánchez L., Campello L., Maneu V., De la Villa P., Lax P., Pinilla I. (2014). Cellular Responses Following Retinal Injuries and Therapeutic Approaches for Neurodegenerative Diseases. Prog. Retin. Eye Res..

[137] Li L., Anand M., Rao K.N., Khanna H. (2015). Cilia in Photoreceptors. Methods Cell Biol..

[138] Bujakowska K.M., Liu Q., Pierce E.A. (2017). Photoreceptor Cilia and Retinal Ciliopathies. Cold Spring Harb. Perspect. Biol..

[139] May-Simera H., Nagel-Wolfrum K., Wolfrum U. (2017). Cilia–The Sensory Antennae in the Eye. Prog. Retin. Eye Res..

[140] Narayan D.S., Chidlow G., Wood J.P.M., Casson R.J. (2017). Glucose Metabolism in Mammalian Photoreceptor Inner and Outer Segments. Clin. Exp. Ophthalmol..

[141] Seo S., Datta P. (2017). Photoreceptor Outer Segment as a Sink for Membrane Proteins: Hypothesis and Implications in Retinal Ciliopathies. Hum. Mol. Genet..

[142] Baehr W., Hanke-Gogokhia C., Sharif A., Reed M., Dahl T., Frederick J.M., Ying G. (2019). Insights Into Photoreceptor Ciliogenesis Revealed by Animal Models. Prog. Retin. Eye Res..

[143] Imanishi Y. (2019). Protein Sorting in Healthy and Diseased Photoreceptors. Annu. Rev. Vis. Sci..

[144] Hayes M.J., Tracey-White D., Kam J.H., Powner M.B., Jeffery G. (2021). The 3D Organisation of Mitochondria in Primate Photoreceptors. Sci. Rep..

