# Supplementary material for: Expression of Dystroglycanopathy-Related Enzymes, POMGNT2 and POMGNT1, in the Mammalian Retina and 661W Cone-like Cell Line
Source: Biomedicines. 2025 Nov 11;13(11):2759. doi: 10.3390/biomedicines13112759 (PMC12650532; doi:10.3390/biomedicines13112759)
Supplement: Supplementary file 1 [file biomedicines-13-02759-s001.zip › biomedicines-3841719-supplementary.pdf]

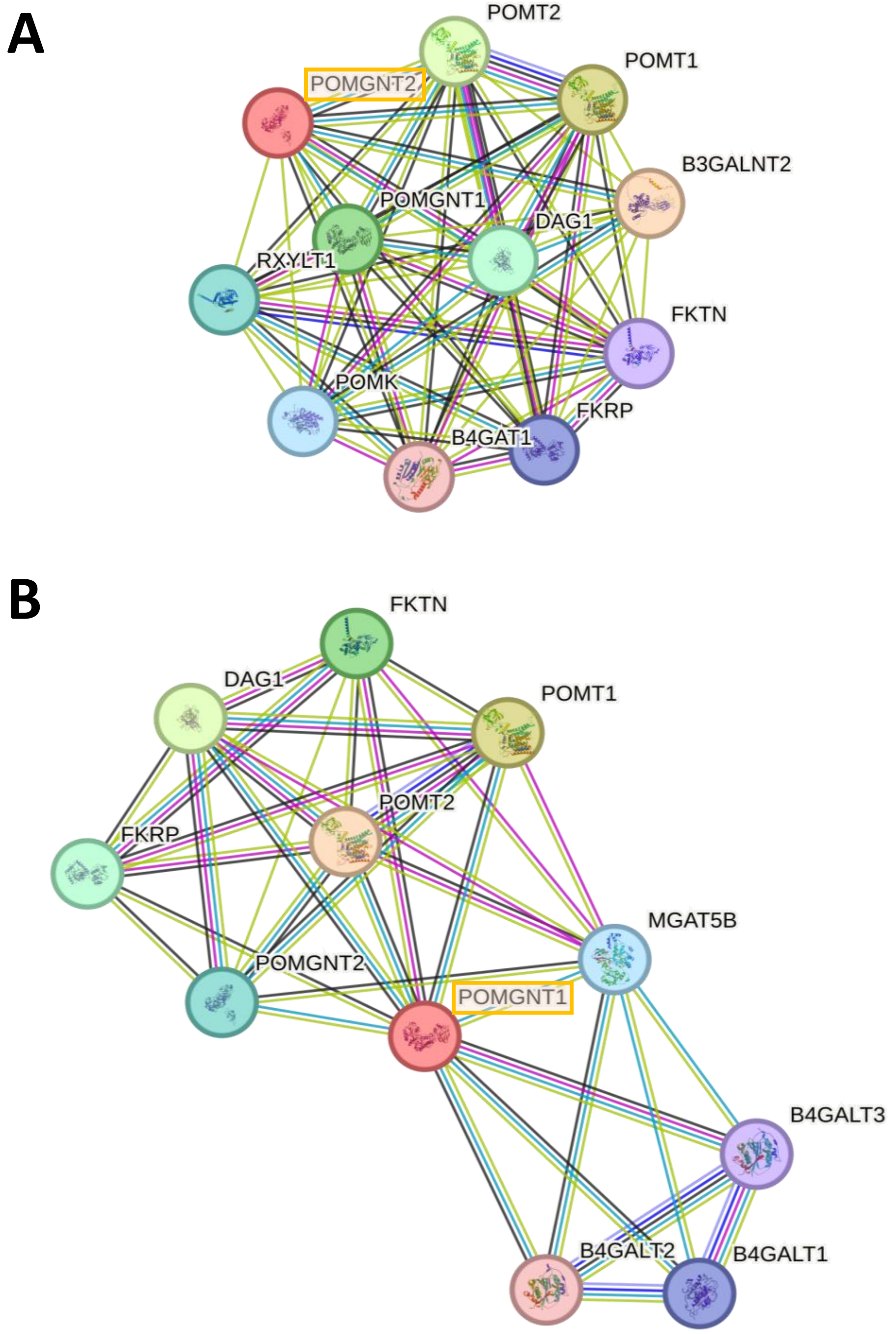

Supplementary Figure S1. Networks of (known and predicted) protein-protein interactions generated by the STRING (v12.0) webtool for POMGNT2 (**A**) and POMGNT1 (**B**) proteins, both boxed in orange.
